# Supplementary material for: South-to-north migration preceded the advent of intensive farming in the Maya region
Source: Nat Commun. 2022 Mar 22;13:1530. doi: 10.1038/s41467-022-29158-y (PMC8940966; doi:10.1038/s41467-022-29158-y)
Supplement: Supplementary file 1 — Supplementary Information [file 41467_2022_29158_MOESM1_ESM.pdf]

Supplementary information

South-to-North Migration Preceded the Advent of Intensive Farming in the Maya Region

In the format provided by the authors and unedited

Douglas J. Kennett*, Mark Lipson*, Keith M. Prufer*, David Mora-Marín, Richard J. George, Nadin Rohland, Mark Robinson, Willa R. Trask, Heather H.J. Edgar, Ethan C. Hill, Erin E. Ray, Paige Lynch, Emily Moes, Lexi O'Donnell, Thomas K. Harper, Emily J. Kate, Josue Ramos, John Morris, Said M. Gutierrez, Timothy M. Ryan, Brendan J. Culleton, Jaime J. Awe, & David Reich*

*corresponding authors

Supplementary Note 1: Site Description and Stratigraphy
(incorporates Supplementary Figures 1-3 in the locations where they are referenced)

All data reported here derive from archaeological excavations at two rock-shelters, Mayahak Cab Pek (MHCP, place of offerings in Q'eqchi' Maya) and Saki Tzul (ST, white mountain in Mopan Maya) conducted in 1998, 2014, and 2016-2018. These shelters are located in the Bladen Nature Reserve (BNR) in an interior valley in the Maya Mountains in southern Belize. The Maya Mountains are a rugged (~400-1000 masl) karst aproned volcanic range that is the largest relief feature in the Maya Lowlands[1]. The region has high precipitation receiving >3,000 mm of seasonally distributed rainfall annually[2]. The BNR is a remote and roadless protected area with little evidence of human impacts over the past 1,000 years. Conducting archaeological research in this area is logistically challenging though its remote location has been a deterrent to looting. MHCP and ST are positioned above active floodplains at ~430 masl along the Bladen Branch of the Monkey River and the Ek Xux Creek, respectively. Neotropical broadleaf forest predominates in the BNR, which provides a range of plant and animal tropical resources native to the region[3,4]. Overall, protein and carbohydrate resources to support human populations are dispersed, relatively low density, and seasonally modulated and could not have supported concentrated human populations without agriculture[5,6]. Within this environment, both MHCP and ST are sheltered from rainfall and contain dry sediments that have favored the preservation of bone and carbonized plant materials. Taphonomic disturbances are minimal and stratigraphy in both shelters is relatively undisturbed[7,8].

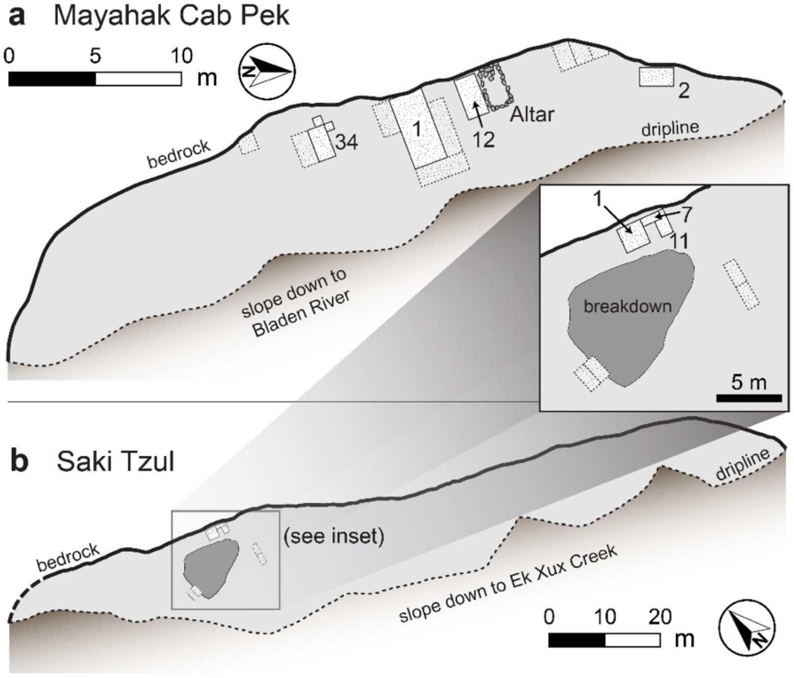

Supplementary Figure 1 | Plan views of MHCP (A) and ST (B) rock-shelters. Excavation units containing individuals included in this study are numbered and shown with solid lines. Other excavations are shown with dotted lines.

MHCP is formed by an east-facing 20 m high limestone outcrop that creates a 26 m wide and 6 m deep rock-shelter with an ENE aspect. The sediments in the shelter are dry and there is limited root activity inside the dripline (~160 m^2). Individuals included in this study were recovered from three excavation units (Supplementary Figure 1). Across all units the stratigraphy is characterized by an ~3.0 m deep sequence of cultural midden and mortuary deposits (Supplementary Figure 2). The lowest stratigraphic units are organic rich (silt to silty-loam) and contain debris from the limestone cliff outcrop, igneous flaked stone tools of local origin (choppers, hammer stones), large chert bifaces (Lowe Points), animal, riverine mollusks (*Pachychilus* spp.), and human remains. These deposits do not contain pottery and date between 12,500 and 4,000 BP.[7] The upper portion of the sequence (< 1 m deep) is comprised of alternating layers of organic rich rocky sediment and sit atop a dense *Pachychilus* spp.(riverine gastropod) midden. These deposits date after ~4,000 BP and contain pottery fragments, flaked stone chert and igneous tools, and the remains of mammals, birds, and reptiles.

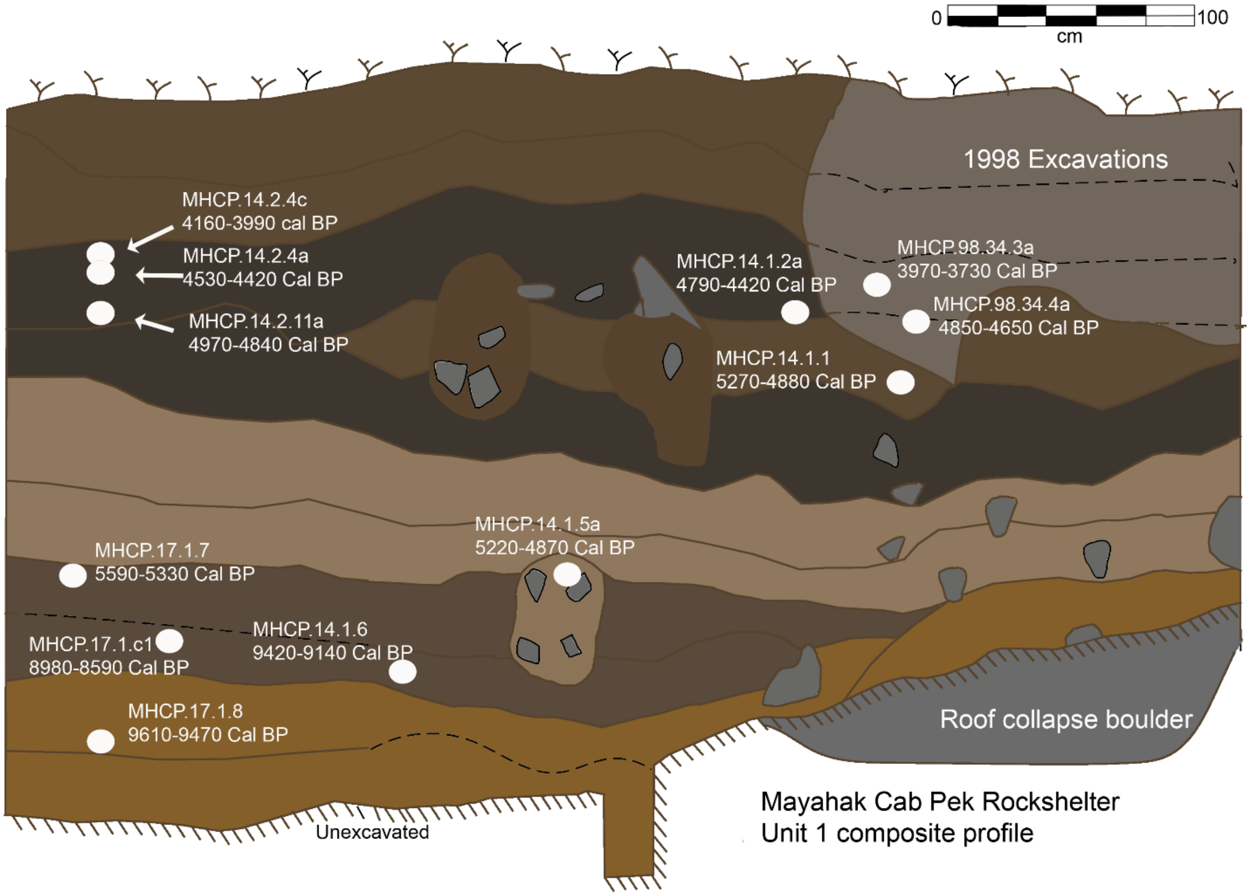

Supplementary Figure 2 | Composite profile drawing of MHCP. Shows relative stratigraphic locations of human remains included in this study.

ST formed below another sheer limestone cliff face that is located 1.4 km to the northeast of MHCP across the Ek Xux valley. The shelter sits 70 m above the river and it is less than 300 m away from the Classic Period Maya center of Ek Xux[8]. It is larger

(145 m long and 8-15 m wide) than MHCP and has ~ 1700 m^2 of dry sediments inside the dripline. Excavations were conducted in only a small area of the rock-shelter, around a large breakdown boulder that fell from the rock-shelter roof prior to Holocene human use. Multiple excavation units reveal a similar stratigraphic sequence to MHCP, also spanning the past 12,500 years (Supplementary Figure 3). Artifact density is high in the upper ceramic bearing strata with high concentrations of animal bone, burned wood, and disarticulated human remains. Two dense *Pachychilus* lenses (>70% shell) occur just below these mixed deposits. The preceramic deposits dating to the Middle Holocene are dominated by dark midden sediments and high concentrations of *Pachychilus* shells, and contain stone tools, bone, carbonized plant material, and human remains. The Early Holocene sediments change to a relatively compact light gray silt and contain reduced, but uniform, concentrations of *Pachychilis* shells, stone tools, carbonized plant material and human remains.

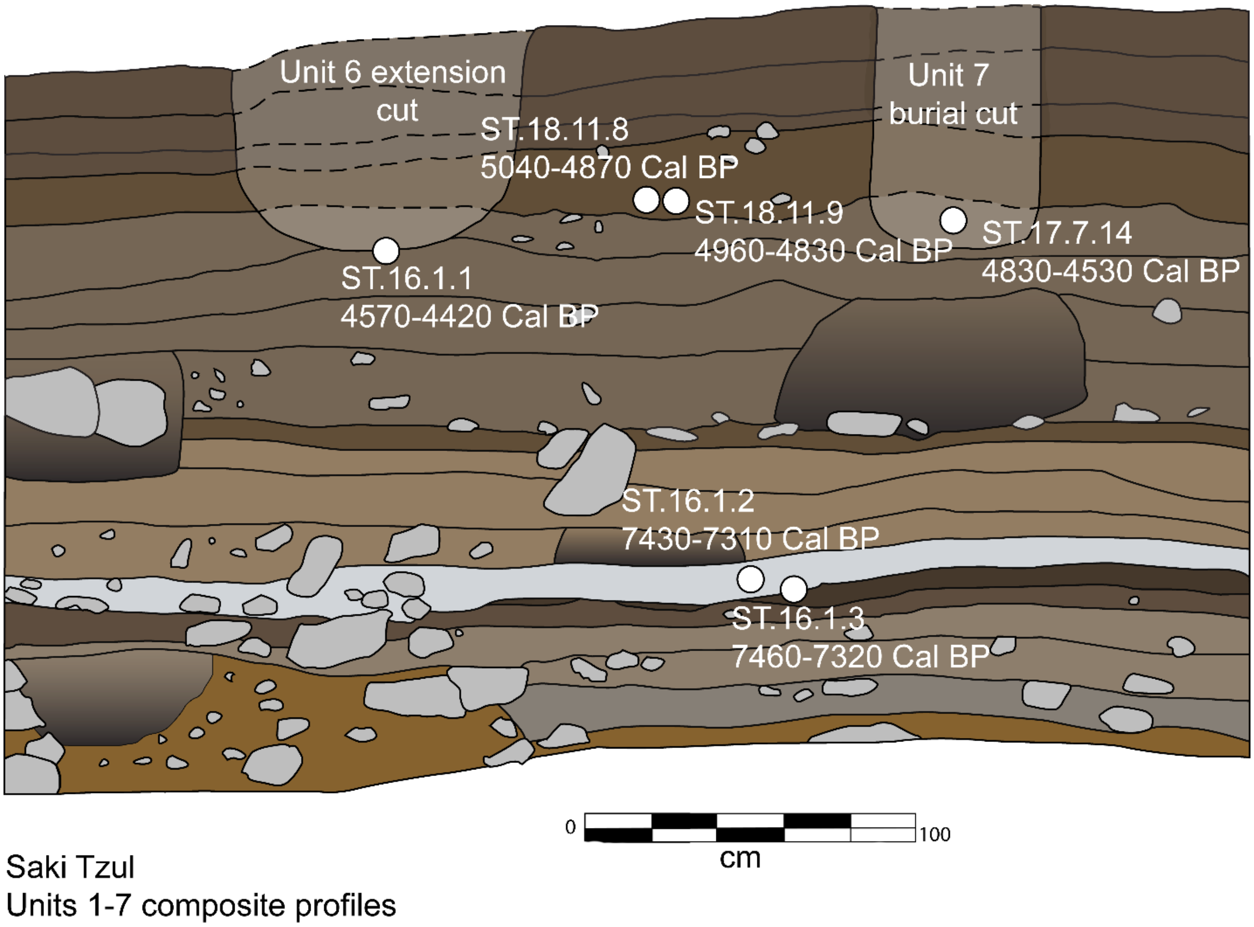

Supplementary Figure 3 | Composite profile drawing of ST. Shows relative stratigraphic locations of human remains included in this study.

Supplementary Note 2: Burial Descriptions & Chronology
(incorporates Supplementary Figures 4-19 in the locations where they are referenced)

Human remains at both rock-shelters reflect similar burial practices and include primary and secondary inhumations, in addition to evidence for the disturbance of earlier burial features by later interments. Isolated elements were recovered from many stratigraphic levels, and while some are the result of either displacement via intrusive disturbance into earlier contexts or bioturbation, others are consistent with intentional secondary deposits of one or more elements. Although a wide range of burial practices are reflected in this long mortuary transect, burials in varying degrees of flexure were the most common and are found in all time periods (Supplementary Figures 4-15). In total, 18 individuals are included in this DNA study. This skeletal population for which we have DNA is represented by 15 males and 9 females, of which 5 are infants, 3 are old adults, 11 are adults, 3 sub-adults, and 2 of indeterminate age (Table S1).

MHCP.98.34.3a: PSUAMS-4292; Harvard Lab ID # I7543 (Fig. S4)

MHCP.98.34.3a consists of the remains of an infant male. Skeletal remains are fragmentary and less than 25% of the skeleton is represented. Bone preservation ranges from fair to poor. MHCP.98.34.3a was buried in a flexed position on its right side, with the head oriented to the north. This individual is associated with isolated elements from a second infant (MHCP.98.34.4b), and an adult.

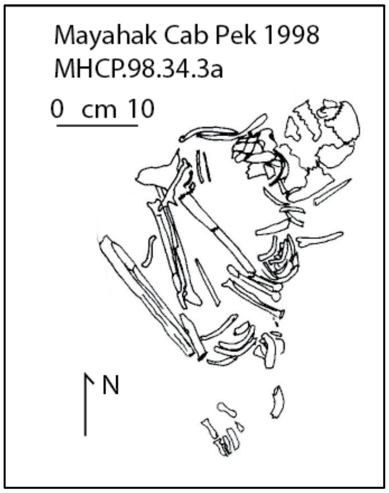

Supplementary Figure 4 | Excavation drawing: MHCP.98.34.3a.

MHCP.98.34.4b: PSUAMS-3774; Harvard Lab ID # I7544

MHCP.98.34.4b is represented by an isolated petrous bone of an infant female recovered from Unit 34 excavated in 1998, directly below MHCP.98.34.3a. This individual was identified during the skeletal analysis of MHCP.98.34.4a and DNA analysis confirmed it to be a distinct individual. Isolated remains; not illustrated.

MHCP.14.1.A5: PSUAMS-6381 (context date); Harvard Lab ID # I20428

MHCP.14.1.A5 is represented by an isolated right temporal bone found in the fill of level 5 from unit 1. It was not found in association with other material. Isolated remains: not illustrated.

MHCP.14.1.1: PSUAMS-2333; UCIAMS-151866; Harvard Lab ID # I5454 (Fig. S5)

MHCP.14.1.1 consists of a middle to late adult male. Bone preservation is poor, and less than 25% of the skeleton is present. The individual was placed in a tightly flexed supine position; the head was oriented to the north and facing east/southeast. MHCP.14.1.1 was recovered with several isolated infant bones which may be associated with remains recovered during the 1998 excavations.

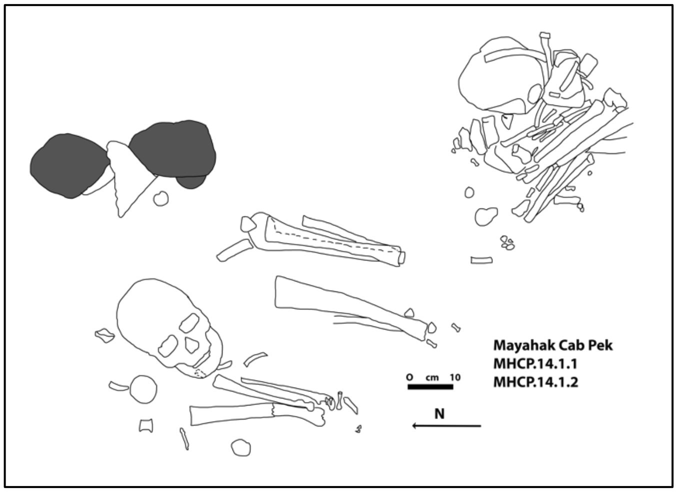

Supplementary Figure 5 | Excavation drawing: MHCP.14.1.1 (left) and MHCP 14.1.2a (right).

MHCP.14.1.2a: PSUAMS-1401; Harvard Lab ID # I6235 (Fig S5)

MHCP.14.1.2a consists of the remains of a subadult male. Bone preservation is poor; bones are highly fragmented, and less than 25% of the skeleton is present. This individual was buried in a very tightly flexed seated position, with the head oriented to the north and facing south. This burial feature contained several isolated adult elements.

MHCP.14.1.5a: PSUAMS-1402; UCIAMS-151853; Harvard Lab ID # I3442 (Fig. S6)

MHCP.14.1.5a consists of a subadult male. Approximately 75% of the skeleton is present, with bone preservation ranging from fair to good. This individual was buried in a tightly flexed position on their left side; the head was oriented to the north and facing east. Isolated remains from at least one additional adult individual were recovered from the burial fill.

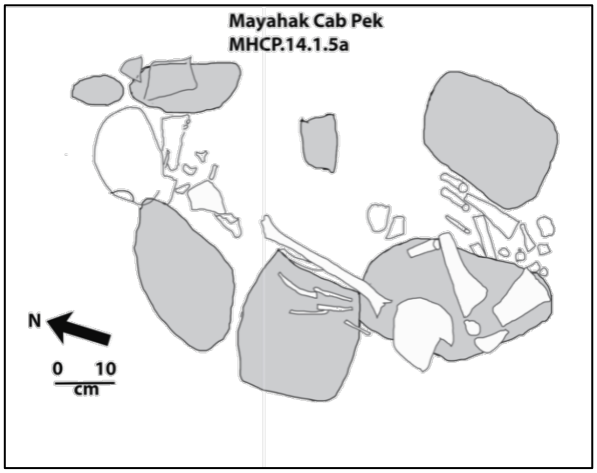

Supplementary Figure 6 | Excavation drawing: MHCP.14.1.5a.

MHCP.14.1.6: UCIAMS-151854; UCIAMS-151855; Harvard Lab ID # I3443 (Fig. S7)

MHCP.14.1.6 consists of a middle to old adult female. Approximately 75% of the skeleton was recovered and is in fair condition. Within the burial feature, the majority of the remains were disarticulated and placed in a tight cluster, although several instances of anatomical articulation were observed. Seven stone tools and 208 pieces of debitage were recovered within ~5 centimeters of the skeleton in the burial feature fill.

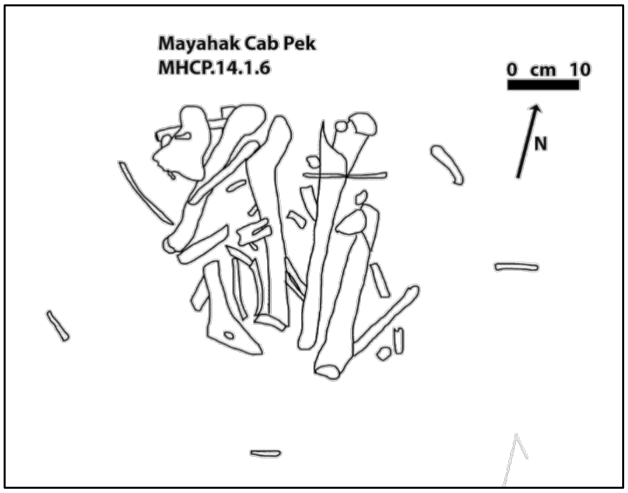

Supplementary Figure 7 | Excavation drawing: MHCP.14.1.6.

MHCP.14.2.4a: UCIAMS-186360; Harvard Lab ID # I5455 (Fig. S8)

MHCP.14.2.4a consists of an adult male. Bone preservation is poor, and most elements have some degree of fragmentation. This individual was buried in an extended supine position, with the head oriented to the north and placed upon an oval-shaped *metate*. Several infant elements (MHCP14.2.4c) were recovered from around the pelvic region. This burial also contained an isolated duplicate adult right radius.

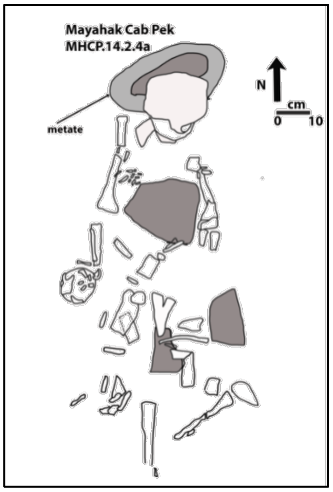

Supplementary Figure 8 | Excavation drawing: MHCP.14.2.4a; note that the cranium is resting on a groundstone metate.

MHCP.14.2.4c: PSUAMS-2681; Harvard Lab ID # I8041

MHCP.14.2.4c consists of remains of an infant male. This individual consists of several infant bones of similar developmental age, all of which were recovered from around the pelvic region of MHCP14.2.4a (see Fig. S8). Burial position could not be determined due to the paucity of remains and poor bone preservation. Partial remains: not illustrated.

MHCP.17.1.7: PSUAMS-3607; Harvard Lab ID # I13267 (Fig. S9)

MHCP.17.1.7 consists of a young adult female. The individual was buried in a tightly flexed, supine position, though slightly turned to the right side; the head was orientated to the north/northwest. The arms are under the knees and the feet were flexed upwards. Approximately 30% of the skeleton is present and bone preservation is poor.

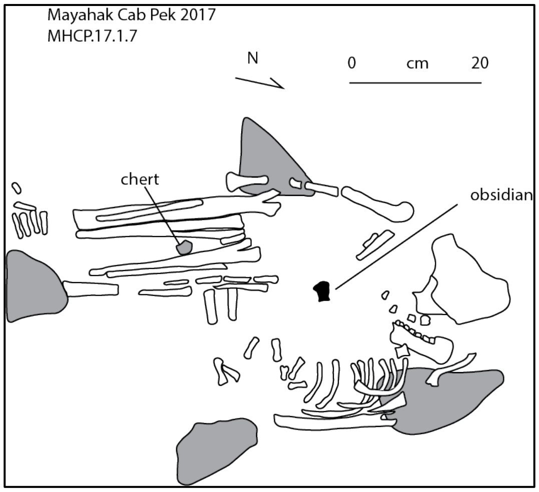

Supplementary Figure 9 | Excavation drawing: MHCP.17.1.7.

MHCP.17.1.8: PSUAMS-4290; Harvard Lab ID # I13268 (Fig. S10)

MHCP.17.1.8 consists of a middle adult male. More than 75% of the skeleton is present, and bone preservation is good. This individual was buried in a semi-flexed position on their right side, with the head oriented in the east and facing south.

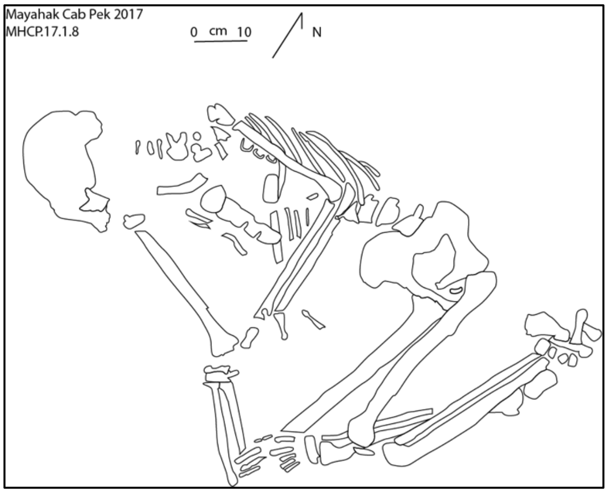

Supplementary Figure 10 | Excavation drawing: MHCP.17.1.8.

MHCP.17.2.11a: PSUAMS-4582; Harvard Lab ID # I19167 (Fig. S11)

MHCP.17.2.11a consists of a middle adult male. Only approximately 30% of the skeleton is present, and bone preservation is poor. The remains were recovered from below a circular rock feature that was originally associated with MHCP.14.2.4a-c. The bones were not recovered in relative anatomical position, suggesting that this may represent a secondary burial feature.

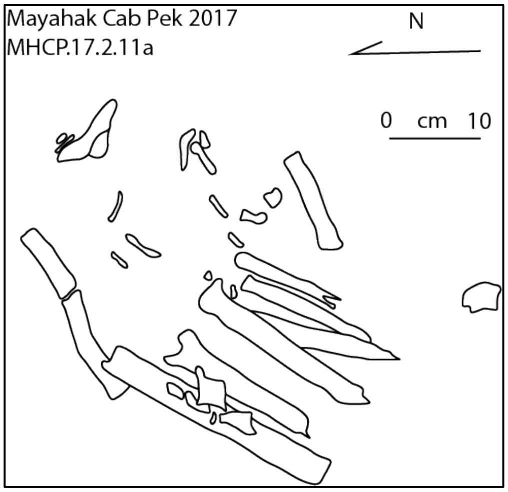

Supplementary Figure 11 | Excavation drawing: MHCP.17.2.11a.

MHCP.17.1.1a: Dated by association with MHCP.17.1.c1; Harvard Lab ID # I19171 (Fig. S12)

MHCP.17.1.1a is represented by a left temporal bone from a female of unknown age. This element was recovered from a mortuary feature containing the commingled remains of a minimum of seven individuals: three adults and four subadults. Individuals MHCP.17.1.a119, MCHP.17.1.c1, and MHCP.17.1.1b were also recovered from this context. No more than 25% of any single individual was recovered from the feature, and all of the recovered elements are fragmentary and fairly preserved. Partial remains: not illustrated.

MHCP.17.1.1b: Dated by association with MHCP.17.1.c1; Harvard Lab ID # I19170 (Fig. S12)

MHCP.17.1.1b is represented by a right temporal bone from a female of unknown age. This element was recovered from a mortuary feature containing the commingled remains of a minimum of seven individuals: three adults and four subadults. Individuals MHCP.17.1.a119, MCHP.17.1.c1, and MHCP.17.1.1a were also recovered from this context. No more than 25% of any single individual was recovered from the feature, and all of the recovered elements are fragmentary and fairly preserved.

MHCP.17.1.c1: PSUAMS-4800; Harvard Lab ID # I19169 (Fig. S12)

MHCP.17.1.c1 is represented by a left temporal bone from an adult male. This element was recovered from a mortuary feature containing the commingled remains of a minimum of seven individuals: three adults and four subadults. Individuals MCHP.17.1.1a, and MHCP.17.1.1b were also recovered from this large diffuse feature containing multiple adults and infants. No more than 25% of any single individual was recovered from the feature, and all the recovered elements are fragmentary and in a fair state of preservation.

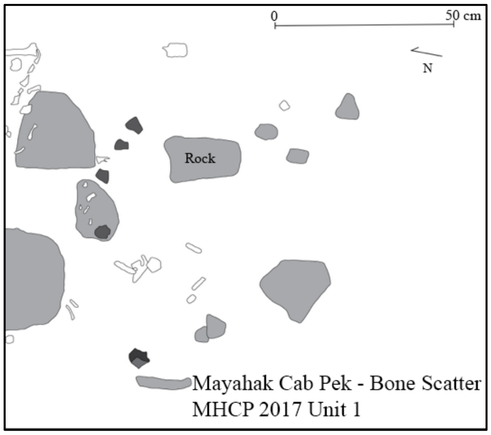

Supplementary Figure 12 | Excavation drawing: MHCP 2017 Unit 1, bone scatter including MHCP.17.1.1a, MHCP.17.1.1b, and MHCP.17.1.c1.

MHCP.19.12.10: PSUAMS-7434 (context date); Harvard Lab ID # I24542 (Fig. S13)

MHCP.19.12.10 consists of the remains of an old adult female. Approximately 50% of the skeleton was recovered and is poorly preserved. This individual was buried

in a tightly flexed position, slightly turned to the left side. The head was in the east, facing south. Materials associated with MHCP.19.12.10 include a chert scraper, and 20-30 lithic flakes and faunal bone fragments.

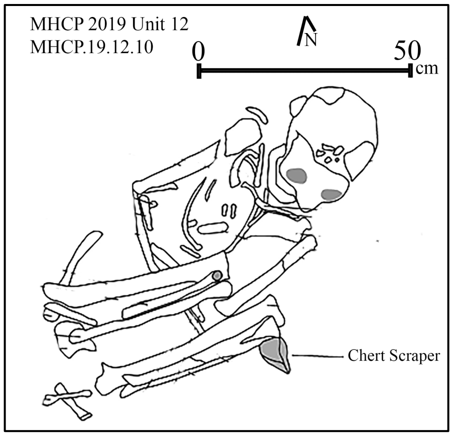

Supplementary Figure 13 | Excavation drawing: MHCP.19.12.10.

MHCP.19.12.17: PSUAMS-8121(context date); Harvard Lab ID # I24541(Fig. S14)

MHCP.19.12.17 consists of the remains of an adult of unknown sex. Approximately 30% of the skeleton is present and is poorly preserved. This individual was buried in a tightly flexed position on their back, slightly rotated to their right. MHCP.19.12.17 was buried on an east-west axis in a space between four large limestone rocks, with the head in the east, (likely) facing south.

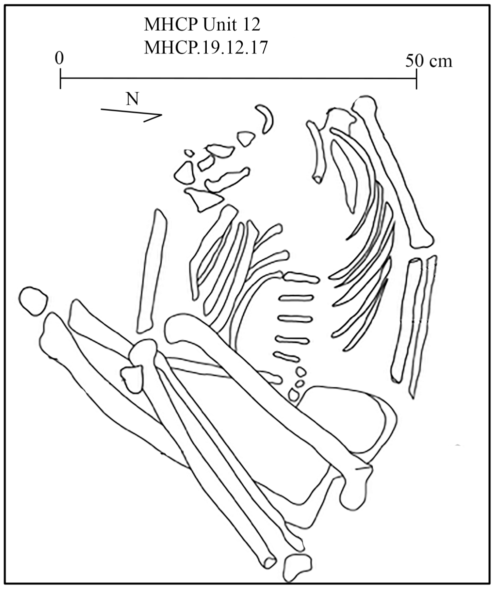

Supplementary Figure 14 | Excavation drawing: MHCP.19.12.17.

MHCP.19.12.18: PSUAMS-7428 (context date); Harvard Lab ID # I24540 (Fig. S15)

MHCP.19.12.18 consists of the remains of an old adult female. Approximately 30% of the skeleton is present; preservation condition is poor. This individual was

buried in a tightly flexed position on a north-south axis, lying on the left side. The head was in the north, facing south.

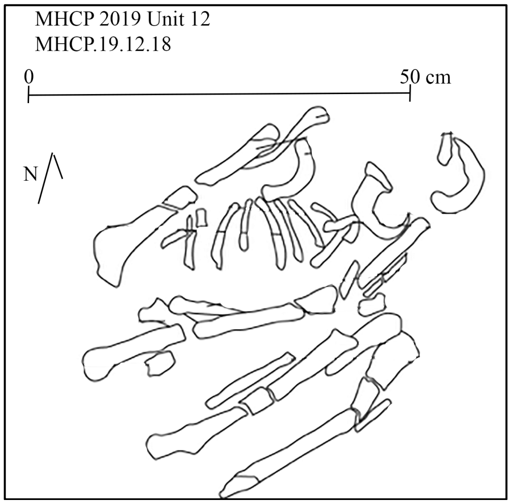

Supplementary Figure 15 | Excavation drawing: MHCP.19.12.18.

ST.16.1.1: PSUAMS-1403; Harvard Lab ID # I6236 (Fig. S16)

ST.16.1.1 consists of the remains of an infant female. More than 75% of the skeleton was recovered and bone preservation is excellent. ST.16.1.1 was buried in a tightly flexed position, on the right side; the head was oriented to the east and facing north. Materials associated with this burial context include groundstone, faunal bone, macro-botanicals, isolated human bone, and obsidian.

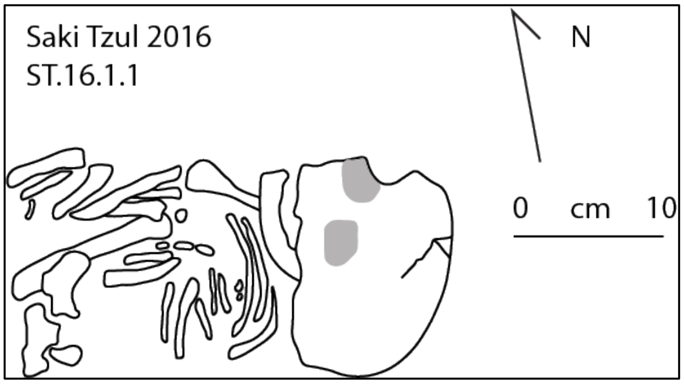

Supplementary Figure 16 | Excavation drawing: ST.16.1.1.

ST.16.1.2: PSUAMS-3205; Harvard Lab ID # I5456 (Fig. S17)

ST.16.1.2 consists of the remains of a middle adult male. Approximately 90% of the skeleton is present and the bone is well-preserved. ST.16.1.2 and ST.16.1.3 were both interred in the same burial feature, with ST.16.1.2 occupying the western portion of the burial feature. The individual was buried in a flexed position on the left side, with the head oriented to the north/northeast and facing east.

ST.16.1.3: PSUAMS-3206; Harvard Lab ID # I5457 (Fig. S17)

ST.16.1.3 consists of the remains of a middle adult male. Approximately 90% of the skeleton is present; bone preservation is excellent. This individual was interred in the same burial feature as ST.16.1.2, occupying the eastern portion of the feature. ST.16.1.3 was placed a flexed position on their right side, with their head oriented to the northeast and neck tightly flexed. The legs of ST.16.1.3 were tightly flexed, and the left arm was extended such that the forearm was resting over the lower torso of ST.16.1.2. The right arm was flexed with the right hand under the pelvis, and directly on top of the tibia of ST.16.1.2.

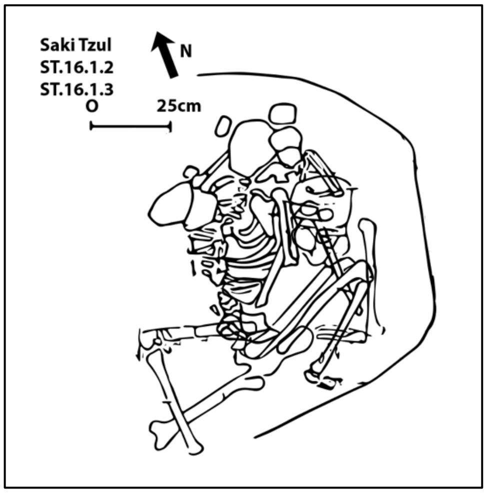

Supplementary Figure 17 | Excavation drawing: ST.16.1.2 and ST.16.1.3 (double burial).

ST.17.7.14: PSUAMS-5127; Harvard Lab ID # I19950 (Fig. S18)

ST.17.7.14 consists of the remains of an infant male. Approximately 75% of the skeleton is present, and the bone is well-preserved. This individual was buried in a flexed position on the right side, with the head oriented to the south and facing east.

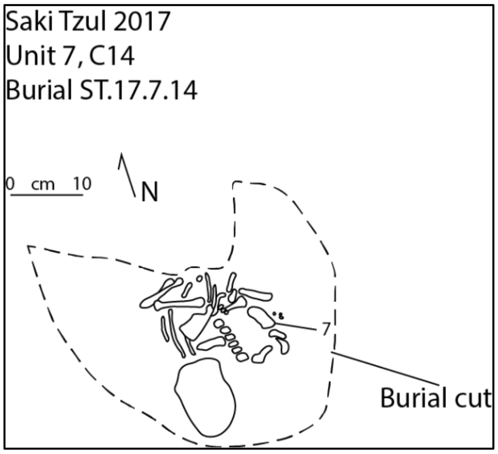

Supplementary Figure 18 | Excavation drawing: ST.17.7.14.

ST.18.11.8: PSUAMS-5896; Harvard Lab ID # I19942 (Fig. S19)

ST.18.11.8 consists of an old adult female. Approximately 85% of the skeleton is present; bone preservation is fair. ST.18.11.8 was buried in a flexed position on the right side, with the head oriented to the south and facing east. This individual was recovered directly north of the burial feature containing ST.18.11.9. Archaeological evidence suggests that ST.18.11.9 was interred first, and later partially impacted during the burial of ST.18.11.8.

ST.18.11.9: PSUAMS-5897; Harvard Lab ID # I19944 (Fig. S19)

ST.18.11.9 consists of a middle adult male. Approximately 80% of the skeleton is present, and bone preservation is fair. This individual was buried in a flexed position on their right side, with the head oriented to the south and facing east. This individual was recovered directly south of the burial feature containing ST.18.11.9. Archaeological evidence suggests that ST.18.11.9 was interred first, with the later interment of ST.18.11.8 disturbing the feet of ST.18.11.9.

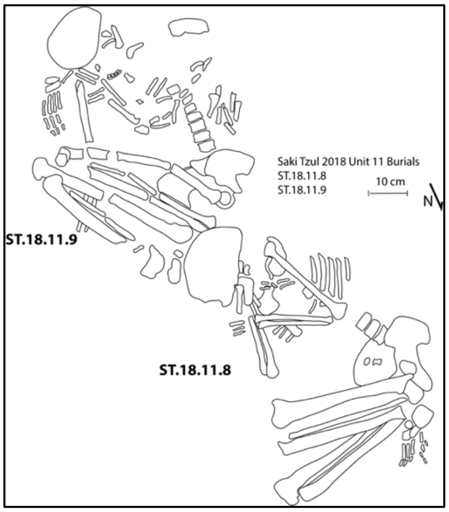

Supplementary Figure 19 | Excavation drawing: ST.18.11.8 and ST.18.11.9.

Supplementary Note 3: Background to Regional Languages

Chibchan is a family of 16 extant (7–8 extinct) languages spoken from northern Venezuela and Colombia to eastern Honduras (Fig. 1)[9]. The highest linguistic diversity of the Chibchan family occurs today in Costa Rica and Panama near the Isthmian land bridge to South America, and this is hypothesized to be the original homeland from which languages diversified from proto-Chibchan (PC) before 5,500 years ago[9]. Mayan is a family of 31 languages spoken across southern Mexico, Guatemala, Belize, and northern Honduras. It is inferred to have diverged from an ancestral proto-Mayan (PM) language around 4,200 years ago, probably in the western Guatemalan highlands based on reconstructed PM terms specific to highland plants and animals[10].

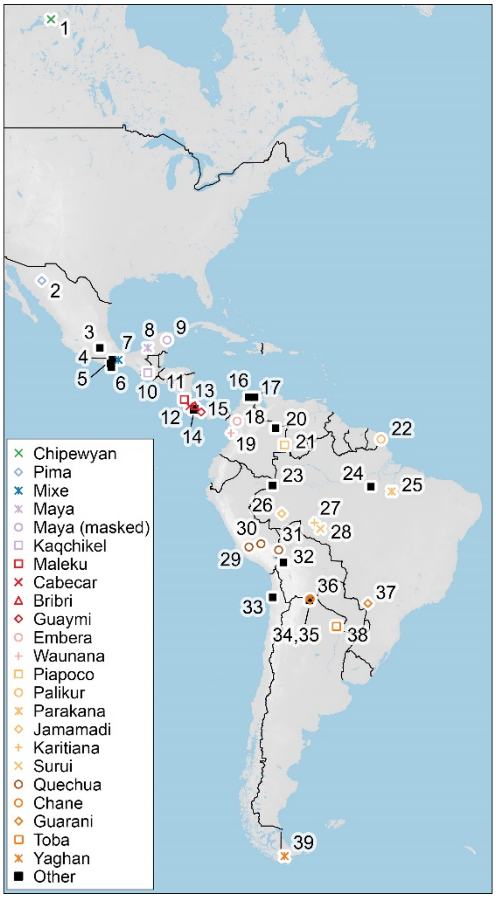

Key	Population	N (total)	Language family	Sampling location	Lat	Long
1	Chipewyan	3	Na-Dene	Canada	59.6	-107.3
2	Pima	14	Uto-Aztecan	Mexico	29.3	-108.8
3	Nahua	2	Uto-Aztecan	Mexico	19.0	-99.1
4	Mixtec	1	Oto-Manguean	Mexico	17.0	-97.0
5	Zapotec	4	Oto-Manguean	Mexico	16.5	-97.2
6	Zapotec	4	Oto-Manguean	Mexico	16.0	-97.0
7	Mixe	9	Mixe-Zoque	Mexico	17.0	-96.0
8	Maya	2	Mayan	Mexico	19.0	-91.0
9	Maya (masked)	17	Mayan	Mexico	20.3	-87.8
10	Kaqchikel	1	Mayan	Guatemala	15.0	-91.0
11	Maleku/Guatuso	2	Chibchan	Costa Rica	10.6	-84.8
12	Cabecar	24	Chibchan	Costa Rica	9.5	-84.0
13	Bribri	2	Chibchan	Costa Rica	9.4	-83.1
14	Teribe	2	Chibchan	Costa Rica	9.0	-83.2
15	Guaymi/Movere	5	Chibchan	Costa Rica	8.5	-82.0
16	Kogi	4	Chibchan	Colombia	11.0	-74.0
17	Wayuu	3	Arawakan	Colombia	11.0	-73.0
18	Embera	5	Chocoan	Colombia	7.0	-76.0
19	Waunana	3	Chocoan	Colombia	5.0	-77.0
20	Guahibo	6	Guahiban	Colombia	5.8	-69.5
21	Piapoco	6	Arawakan	Colombia	3.0	-68.0
22	Palikur	2	Arawakan	Guiana	4.0	-51.8
23	Ticuna	5	(isolate)	Colombia	-3.8	-70.0
24	Arara	1	Cariban	Brazil	-4.0	-53.5
25	Parakana	1	Tupian	Brazil	-4.8	-50.0
26	Jamamadi	1	Arawan	Brazil	-8.5	-68.5
27	Karitiana	13	Tupian	Brazil	-10.0	-63.0
28	Surui	24	Tupian	Brazil	-11.0	-62.0
29	Quechua	3	Quechua	Bolivia & Peru	-14.0	-74.0
30	Quechua	3	Quechua	Bolivia & Peru	-13.5	-72.0
31	Quechua	3	Quechua	Bolivia & Peru	-14.5	-69.0
32	Aymara	4	Aymaran	Bolivia & Chile	-16.5	-68.2
33	Aymara	4	Aymaran	Bolivia & Chile	-22.0	-70.0
34	Wichi	4	Matacoan	Argentina	-22.5	-63.8
35	Guarani	3	Tupian	Paraguay & Argentina	-22.5	-63.8
36	Chane	2	Arawakan	Argentina	-22.3	-63.7
37	Guarani	3	Tupian	Paraguay & Argentina	-23.0	-54.0
38	Toba	2	Guaicuruan	Argentina	-26.5	-59.3
39	Yaghan	1	(isolate)	Chile	-55.0	-68.0

Supplementary Figure 20 | Comparative genome-wide data from present-day individuals. Population names, locations, and language families for present-day populations used in genetic analyses. Symbols match those displayed in PCA (Fig. 2, Supplementary Fig. 23).

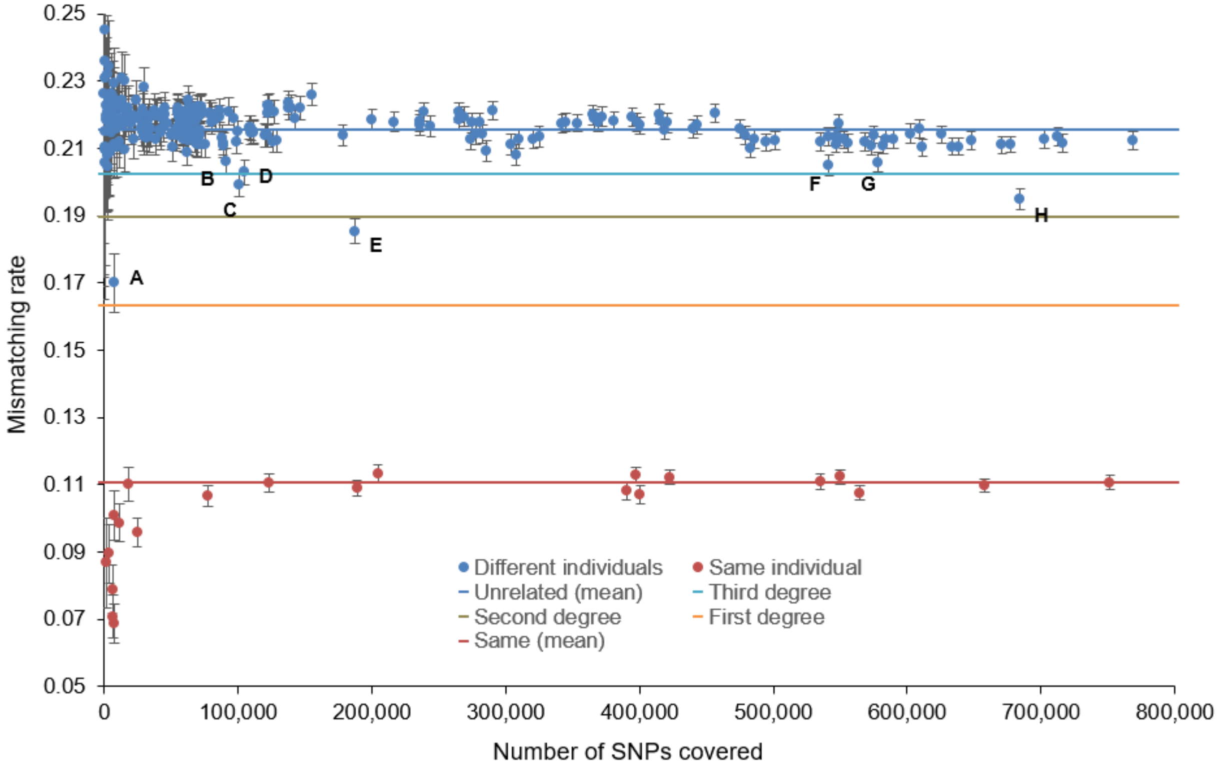

Supplementary Figure 21 | Kinship analysis. Observed same-individual allelic mismatch rates (red points) are approximately one-half those of different-individual comparisons (blue points), as expected for unrelated individuals (computations using very few SNPs are less reliable and precise). Red and dark blue lines (rates of 0.110 and 0.216, respectively) give empirical averages of the points with > 100k SNPs. First-degree relatives are expected to have rates on average halfway between same-individual and unrelated (orange line), second-degree relatives halfway between first-degree and unrelated (brown line), and third-degree halfway between second-degree and unrelated (light blue). Based on the proportion of the genome shared identical by descent (IBD) at different kinship levels, first-degree relatives are expected to have rates on average halfway between same-individual and unrelated (orange line), second-degree relatives halfway between first-degree and unrelated (brown line), and third-degree halfway between second-degree and unrelated (light blue)[11]. Likely and possible relative pairs labeled are as follows: A, MHCP.17.1.c1/MHCP.17.1.1b (first-degree); B, MHCP.98.34.4b/MHCP.98.34.3a (possible, third or greater); C, MHCP.14.1.2a/ MHCP.98.34.3a (possible, third or greater); D, ST.16.1.1/MHCP.98.34.3a (possible, third or greater); E, ST.16.1.3/ST.16.1.2 (second-degree); F, MHCP.17.1.7/ MHCP.19.12.10 (possible, third or greater); G, MHCP.98.34.4b/MHCP.14.1.2a (possible, third or greater); H, MHCP.14.1.2a/ST.16.1.1 (second/third-degree). Bars show two standard errors in each direction around the mean over all available SNPs, with the number of SNPs given on the x-axis for each point.

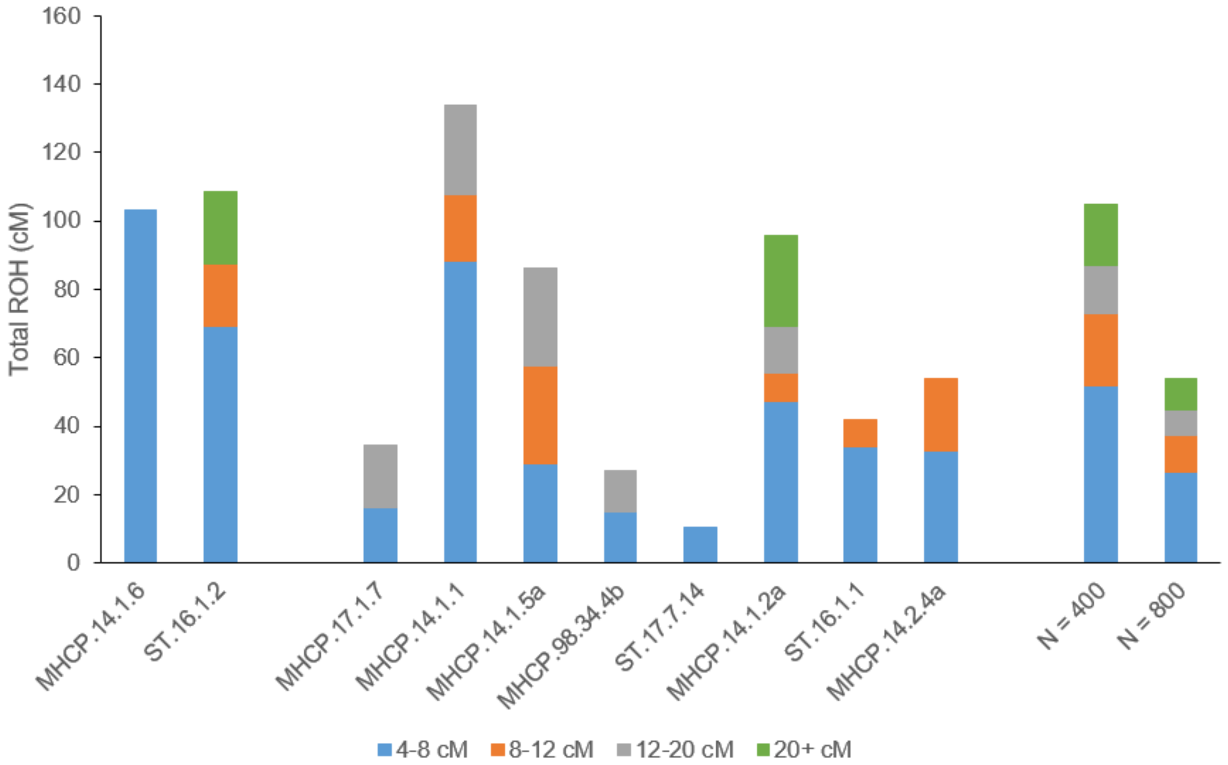

Supplementary Figure 22 | Runs of Homozygosity (ROH). For each individual with sufficient coverage (> ~0.4x), we show total genome-wide lengths of long (> 4 cM [centiMorgans]) inferred ROH segments, subdivided by length bins (colors). Individuals are sorted by date (earliest on the left, with two from 9,600-7,300 cal. BP and eight from 5,600-3,700 cal. BP). A large number of moderate segments (~4-8 cM) indicates a low recent ancestral effective population size, while a large number of very long segments (> 20 cM, not observed here) indicates children of close-kin unions (e.g., first cousin marriages). Expected ROH for example values of effective population size (N) are shown to the right.

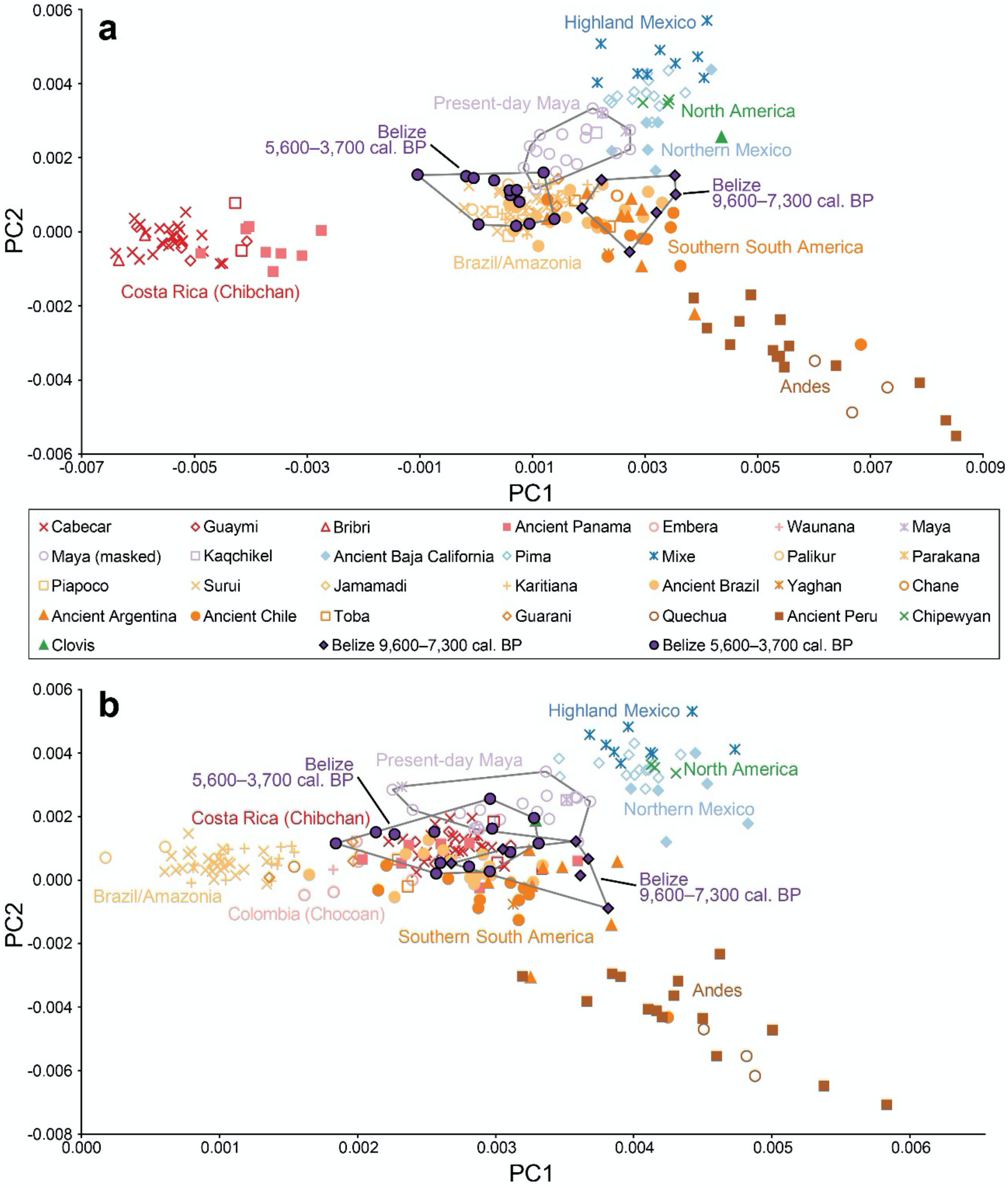

Supplementary Figure 23 | Alternative PCA plots. In the set of populations used to compute the axes, we replaced Chibchan populations with (a) Waunana (5 individuals) or (b) Piapoco (6 individuals). The relative positions of the 9,600-7,300 cal. BP individuals, 5,600-3,700 cal. BP individuals, and present-day Maya are similar across all three versions. The leftward shift of the 5,600-3,700 cal. BP individuals becomes less pronounced in (a) and especially in (b) as compared to Fig. 2a, as expected if they harbor ancestry related to the ancestors of Chibchan populations. We also note that in (b), when Chibchan populations are not specifically related to any groups used to compute the axes, they fall close to the 5,600-3,700 cal. BP individuals.

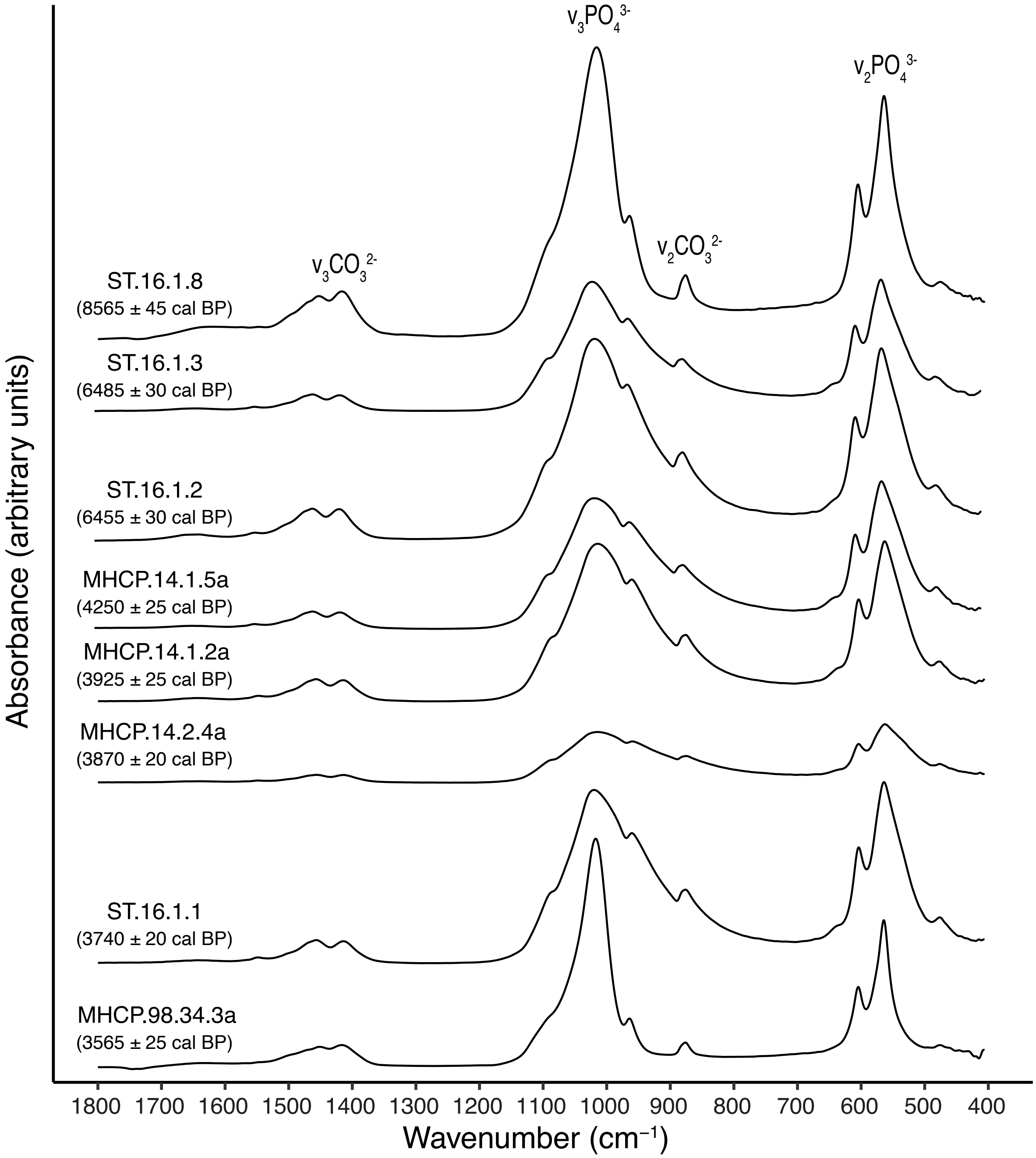

Supplementary Figure 24 | ATR-FTIR spectra of 8 enamel carbonate radiocarbon samples between the 400 and 1,800 cm^{-1} range in the apatite domain. Phosphate (V_2,V_3 PO_4^{3-}) and carbonate (V_2,V_3 CO_3^{2-}) peaks are displayed and were used to evaluate chemical pretreatment and enamel diagenesis. The crystallinity peaks are consistent with ranges observed in archaeological samples with low diagenetic alterations to the mineral components[12]. We note that MHCP.14.2.4a displays a lower overall intensity in the FTIR spectrum.

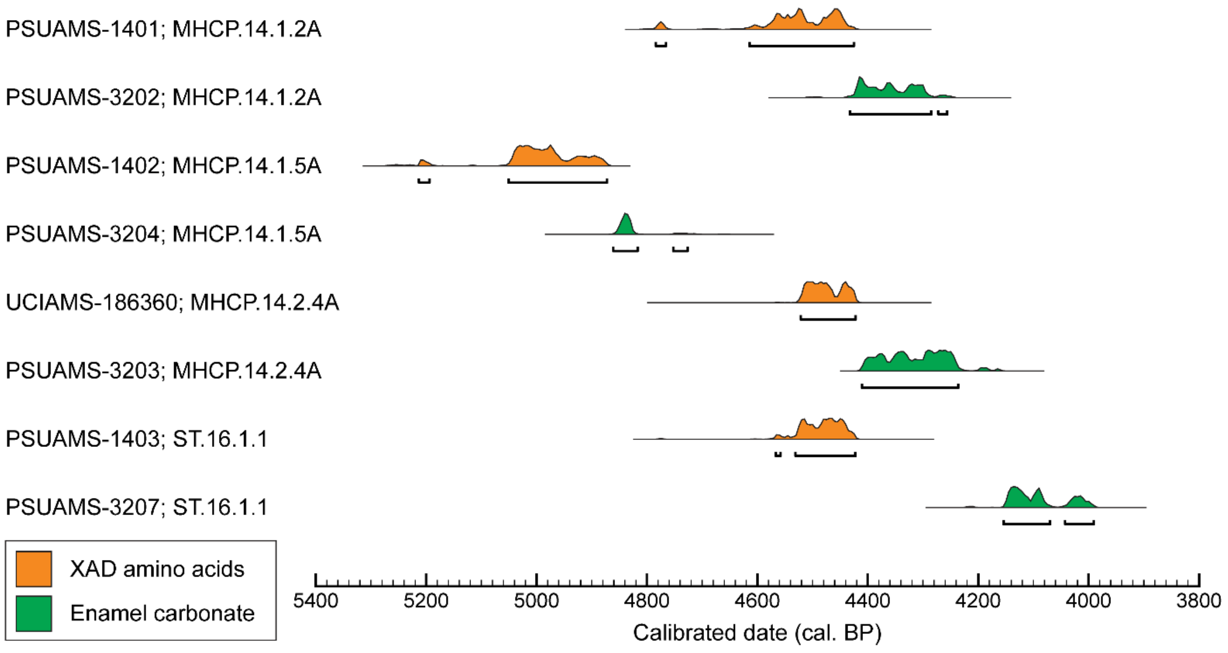

Supplementary Figure 25 | AMS ^{14}C results from four samples with both XAD amino acid and enamel carbonate dates. We observe that enamel carbonate dates average approximately 175 14C years younger than comparable XAD amino acid dates taken from the same sample material.

Supplementary Table 1 | Summary of burials from this study.

Burial ID	Harvard lab ID	Sex (DNA)	Sex (osteology)	Age category	Est. age (yrs)	Burial position	Burial orientation	Percent complete	Preservation condition
MHCP.17.1.8	I13268	M	M	middle adult	31–50	Semi-flexed on right side	head to the E, facing S	>75	good
MHCP.14.1.6	I3443*	F	F	middle-old adult	—	disarticulated	—	~75	fair
ST.16.1.3	I5457*	M	M	middle adult	35–55	flexed laying on right side; neck tightly flexed with head	head to the NE, facing into chest	>75	good–excellent
MHCP.17.1.C1	I19169	M	—	Infant	—	commingled context	—	<25	fair
MHCP.17.1.1A	I19171	F	—	unknown	—	commingled context	—	<25	fair
MHCP.17.1.1B	I19170	F	—	adult	—	commingled context	—	<25	fair
MHCP.19.12.10	I24542	F	F	old adult	—	tightly flexed, laying on left side	head in the E, facing S	~50	poor
MHCP.19.12.17	I24541	M	M	adult	—	tightly flexed, supine, slightly turned on right side	Head in E, facing S	~30	poor
MHCP.19.12.18	I24540	F	F	old adult	—	tightly flexed, laying on left side	Head to the N, facing S	~30	poor
ST.16.1.2	I5456*	M	M	middle adult	35–45	flexed, laying on left side	head to the N/NE facing E	>75	good–excellent
MHCP.17.1.7	I13267	F	F	young adult	—	tightly flexed, supine, slightly turned on right side	head to the N, facing W	~30	poor
MHCP.14.1.A5	I20428	M	—	unknown	—-	Isolated element	—	—	good
MHCP.14.1.1	I5454	M	—	middle-old adult	—	tightly flexed, supine, with knees and arms flexed over the chest	head to the N, facing E/SE	<25	poor
MHCP.14.1.5A	I3442	M	—	subadult	11–15	tightly flexed feet under pelvis on left side	head to the N, facing E	75	fair–good
MHCP.14.1.2A	I6235	M	—	subadult	16–20	tightly flexed, in a seated position	head to the N, facing S	<25	poor
ST.16.1.1	I6236	F	—	subadult	2–3	tightly flexed, laying on right side	head to the E, facing N	>75	excellent
ST.17.7.14	I19950	M	—	infant	4.5–7.5 mo	tightly flexed, laying on right side	head to the S, facing E	~75	good
ST.18.11.9	I19944	M	M	middle adult	40–50	flexed, laying on right side; arms flexed, hands near face	head to the S, facing E	>75	fair
ST.18.11.8	I19942	F	F	old adult	60+	flexed, laying on right side	head to the S, facing E	>75	fair
MHCP.14.2.4A	I5455	M	M	adult	—	extended supine	head to the N	<25	poor
MHCP.14.2.4C	I8041	M	—	infant	<6 mo	indeterminate	—	<25	poor
MHCP.17.2.11A	I19167	M	—	middle adult	35–50	possible secondary	—	<30	poor
MHCP.98.34.4B	I7544	F	—	infant	<1 yr	isolated element	—	<5	poor
MHCP.98.34.3A	I7543	M	—	infant	9–12 mo	flexed, laying on right side	Head to the N	<25	poor–fair

* Previously published [13]

Supplementary Table 2 | Radiocarbon dates for samples with genome-wide data.

^{14}C ID	Harvard lab ID	Burial ID	Material	Process	^{14}C BP	±	C:N	cal. BP (μ)	cal. BP (2σ lo)	cal. BP (2σ hi)
PSUAMS-8121	I24541	MHCP.19.12.17	charcoal (context)	ABA	10165	50	—	11797	11970	11410
PSUAMS-4290	I13268	MHCP.17.1.8	tooth, molar	enamel carbonate	8565	45	—	9535	9660	9470
UCIAMS-151854	I3443	MHCP.14.1.6	bone, femur	XAD	8310	35	3.35	9325	9445	9140
UCIAMS-151855	I3443	MHCP.14.1.6	bone, femur	XAD	8270	35	3.40	9260	9420	9125
PSUAMS-4800	I19169	MHCP.17.1.c1	bone, temporal	XAD	7900	35	3.47	8735	8980	8595
see I19169	I19170	MCHP.17.1.1b	(association with I19169)	—	7900	35	—	8735	8980	8595
PSUAMS-3206	I5457	ST.16.1.3	tooth enamel	enamel carbonate	6485	30	—	7375	7460	7320
PSUAMS-3205	I5456	ST.16.1.2	tooth enamel	enamel carbonate	6455	30	—	7370	7430	7310
PSUAMS-6381	I20428	MHCP.14.1.A5	charcoal (context)	ABA	6090	25	—	6960	7155	6860
PSUAMS-7428	I24540	MHCP.19.12.18	charcoal (context)	ABA	4875	25	—	5610	5660	5490
UCIAMS-228026	I13267	MHCP.17.1.7	bone, temporal	UF	4775	45	3.28	5500	5595	5330
PSUAMS-3607	I13267	MHCP.17.1.7	bone	UF	4725	25	3.26	5450	5580	5325
PSUAMS-2333	I5454	MHCP.14.1.1	bone, temporal	XAD	4430	20	3.34	5040	5270	4880
PSUAMS-1402	I3442	MHCP.14.1.5A	bone, temporal	XAD	4415	25	3.26	5000	5260	4870
PSUAMS-5896	I19942	ST.18.11.8	bone, femur	XAD	4390	20	3.33	4945	5040	4870
PSUAMS-4582	I19167	MHCP.17.2.11A	bone, humerus	XAD	4330	20	3.38	4890	4960	4845
PSUAMS-5897	I19944	ST.18.11.9	bone, femur	XAD	4300	30	3.21	4870	4960	4830
UCIAMS-228020	I7544	MHCP.98.34.4B	bone, temporal	XAD	4210	20	3.22	4760	4845	4650
PSUAMS-7434	I24542	MHCP.19.12.10	charcoal (context)	ABA	4190	25	—	4731	4840	4620
PSUAMS-5127	I19950	ST.17.7.14	bone, humerus	XAD	4125	30	3.41	4675	4820	4530
PSUAMS-1401	I6235	MHCP.14.1.2A	bone, temporal	XAD	4050	30	3.32	4525	4785	4420
PSUAMS-1403	I6236	ST.16.1.1	bone, temporal	XAD	4025	25	3.29	4480	4570	4420
UCIAMS-186360	I5455	MHCP.14.2.4A	tooth, molar	XAD	4005	20	3.29	4470	4525	4420
PSUAMS-2681	I8041	MHCP.14.2.4C	bone, radius	XAD	3735	20	3.31	4080	4155	3990
PSUAMS-4292	I7543	MHCP.98.34.3A	tooth, canine	enamel carbonate	3565	25	—	3860	3965	3730

Supplementary Table 3 | Shown are the top 15 most significant statistics of the form f_4(9,500–7,300 cal. BP, 5,600–3,700 cal. BP; Present-day1, Present-day2) and f_4(5,600–3,700 cal. BP, Present-day Maya; Present-day1, Present-day2), with Z-scores for differences from zero. Single asterisks denote Chibchan populations (top half), and double asterisks denote highland Mexican populations (bottom half). Full lists of results can be found in Supplementary Data 5–6.

Population A	Population B	Population C	Population D	Value	Z-score
9,600-7,300 BP	5,600-3,700 BP	Chipewyan	Kaqchikel	0.001979	4.536
9,600-7,300 BP	5,600-3,700 BP	Chipewyan	Bribri*	0.001828	4.588
9,600-7,300 BP	5,600-3,700 BP	Aymara	Kaqchikel	0.001807	4.609
9,600-7,300 BP	5,600-3,700 BP	Chipewyan	Guaymi*	0.001721	5.02
9,600-7,300 BP	5,600-3,700 BP	Chipewyan	Teribe*	0.001697	4.367
9,600-7,300 BP	5,600-3,700 BP	Aymara	Bribri*	0.001653	4.857
9,600-7,300 BP	5,600-3,700 BP	Chipewyan	Cabecar*	0.001646	4.935
9,600-7,300 BP	5,600-3,700 BP	Chipewyan	Waunana	0.001621	4.647
9,600-7,300 BP	5,600-3,700 BP	Aymara	Guaymi*	0.001547	5.1
9,600-7,300 BP	5,600-3,700 BP	Aymara	Teribe*	0.001522	4.477
9,600-7,300 BP	5,600-3,700 BP	Aymara	Cabecar*	0.001471	5.255
9,600-7,300 BP	5,600-3,700 BP	Aymara	Waunana	0.001447	4.76
9,600-7,300 BP	5,600-3,700 BP	Quechua	Cabecar*	0.001267	4.297
9,600-7,300 BP	5,600-3,700 BP	Mixe	Guaymi*	0.001194	4.482
9,600-7,300 BP	5,600-3,700 BP	Mixe	Cabecar*	0.001118	4.542
5,600-3,700 BP	Present-day Maya	Waunana	Zapotec**	0.001799	6.058
5,600-3,700 BP	Present-day Maya	Waunana	Mixe**	0.001689	6.658
5,600-3,700 BP	Present-day Maya	Guarani	Zapotec**	0.001677	5.732
5,600-3,700 BP	Present-day Maya	Guarani	Mixe**	0.001569	6.777
5,600-3,700 BP	Present-day Maya	Ticuna	Zapotec**	0.001518	5.079
5,600-3,700 BP	Present-day Maya	Cabecar	Zapotec**	0.001466	5.104
5,600-3,700 BP	Present-day Maya	Teribe	Mixe**	0.00143	5.241
5,600-3,700 BP	Present-day Maya	Ticuna	Mixe**	0.001407	5.857
5,600-3,700 BP	Present-day Maya	Wayuu	Mixe**	0.001374	5.929
5,600-3,700 BP	Present-day Maya	Toba	Mixe**	0.001354	5.388
5,600-3,700 BP	Present-day Maya	Cabecar	Mixe**	0.001353	6.203
5,600-3,700 BP	Present-day Maya	Guahibo	Mixe**	0.001323	5.95
5,600-3,700 BP	Present-day Maya	Karitiana	Mixe**	0.001284	5.623
5,600-3,700 BP	Present-day Maya	Embera	Mixe**	0.001199	5.191
5,600-3,700 BP	Present-day Maya	Piapoco	Mixe**	0.001105	5.064

Supplementary Table 4 | Individual-level statistics of the form f_4(Outgroup, Ancient individual; Aymara, Chibchan) using Han Chinese (CHB) as the outgroup and Guaymi, Maleku, and Bribri together to represent Chibchan populations. The horizontal line divides the 9,600-7,300 BP and 5,600-3,700 BP subgroups.

Population A	Population B	Population C	Population D	Value	Z-score
CHB	MHCP.17.1.8	Aymara	Chibchan	0.000517	0.933
CHB	MHCP.14.1.6	Aymara	Chibchan	-0.000707	-1.592
CHB	MHCP.17.1.c1	Aymara	Chibchan	-0.000638	-0.883
CHB	MHCP.17.1.1b	Aymara	Chibchan	-0.000425	-0.58
CHB	ST.16.1.3	Aymara	Chibchan	-0.000387	-0.776
CHB	ST.16.1.2	Aymara	Chibchan	-0.0003	-0.66
CHB	MHCP.19.12.18	Aymara	Chibchan	0.000697	1.479
CHB	MHCP.17.1.7	Aymara	Chibchan	0.000836	1.949
CHB	MHCP.14.1.1	Aymara	Chibchan	0.001623	3.591
CHB	MHCP.14.1.5a	Aymara	Chibchan	0.001458	3.494
CHB	ST.18.11.8	Aymara	Chibchan	0.001281	2.101
CHB	MHCP.98.34.4b	Aymara	Chibchan	0.000787	1.783
CHB	MHCP.19.12.10	Aymara	Chibchan	0.001119	1.956
CHB	ST.17.7.14	Aymara	Chibchan	0.000936	2.127
CHB	MHCP.14.1.2a	Aymara	Chibchan	0.00101	2.335
CHB	ST.16.1.1	Aymara	Chibchan	0.001589	3.898
CHB	MHCP.14.2.4a	Aymara	Chibchan	0.000981	2.274
CHB	MHCP.14.2.4c	Aymara	Chibchan	0.001497	1.955
CHB	MHCP.98.34.3a	Aymara	Chibchan	0.000664	1.103

Supplementary Table 5 | Additional f_4-statistics f_4(A, B; C, D). From top to bottom, the four sections show (1) allele-sharing between the 5,600–3,700 cal. BP individuals and diverse South Americans; (2) allele-sharing between Chibchan populations and South Americans; (3) allele-sharing between present-day Maya and northern Mexicans (Pima); and (4) allele-sharing with Chibchan populations for present-day Maya but not highland Mexicans. Sections (2) and (4) use Han Chinese (CHB) as an outgroup and Guaymi, Maleku, and Bribri together to represent Chibchan populations. Full lists of results from which (1) and (3) are taken can be found in Supplementary Tables 3 and 6.

Population A	Population B	Population C	Population D	Value	Z-score
9,600–7,300 BP	5,600–3,700 BP	Aymara	Piapoco	0.001073	4.056
9,600–7,300 BP	5,600–3,700 BP	Chipewyan	Piapoco	0.001247	3.976
9,600–7,300 BP	5,600–3,700 BP	Aymara	Surui	0.001106	3.863
9,600–7,300 BP	5,600–3,700 BP	Chipewyan	Surui	0.001281	3.807
9,600–7,300 BP	5,600–3,700 BP	Aymara	Guarani	0.000995	3.529
9,600–7,300 BP	5,600–3,700 BP	Chipewyan	Guarani	0.001168	3.513
9,600–7,300 BP	5,600–3,700 BP	Aymara	Waunana	0.001447	4.76
9,600–7,300 BP	5,600–3,700 BP	Chipewyan	Waunana	0.001621	4.647
CHB	Chibchan	Aymara	Piapoco	0.00047	1.67
CHB	Chibchan	Aymara	Surui	0.00085	2.66
CHB	Chibchan	Aymara	Guarani	0.0007	2.4
CHB	Chibchan	Aymara	Waunana	0.00434	13.78
5,600–3,700 BP	Present-day Maya	Waunana	Pima	0.000877	3.414
5,600–3,700 BP	Present-day Maya	Guarani	Pima	0.000757	3.106
5,600–3,700 BP	Present-day Maya	Ticuna	Pima	0.000595	2.316
CHB	Present-day Maya	Aymara	Chibchan	0.00085	2.97
CHB	Mixe	Aymara	Chibchan	-0.00012	-0.48
CHB	Zapotec	Aymara	Chibchan	0.00022	0.84

Supplementary Table 6 | Two-way *qpAdm* model results. Prop. 1/2: proportions of ancestry related (perhaps deeply) to sources 1/2; S.E., standard error. A p-value above a given threshold (e.g., 0.05) indicates a lack of evidence against the model (i.e., a good fit). The p-value from *qpAdm* is computed using Hotelling's T^2 test.

Test	Source 1	Source 2	p-value	Prop. 1	Prop. 2	S.E.
5,600–3,700 BP	9,600–7,300 BP	Guaymi+Maleku+Bribri	0.96	31.20%	68.80%	8.80%
5,600–3,700 BP	9,600–7,300 BP	Guaymi	0.9	33.50%	66.50%	9.30%
5,600–3,700 BP	9,600–7,300 BP	Maleku	0.24	41.70%	58.30%	11.70%
5,600–3,700 BP	9,600–7,300 BP	Bribri	0.98	23.10%	76.90%	13.20%
Present-day Maya	5,600–3,700 BP	Mixe+Zapotec	0.25	75.10%	24.90%	9.60%
Present-day Maya	5,600–3,700 BP	Mixe	0.24	75.90%	24.10%	9.60%
Present-day Maya	5,600–3,700 BP	Zapotec	0.26	74.10%	25.90%	10.10%
Present-day Maya (masked)	5,600–3,700 BP	Mixe+Zapotec	0.01	85.40%	14.60%	7.10%

Supplementary Table 7 | Evidence for (left) regular sound correspondences among basic vocabulary items. Potential links between PM and PC include 9 recurring sound correspondences involving consonants and 9 roots with interlocking sets of recurring biconsonantal sound correspondences.

Proto-Mayan	Proto-Chibchan	Recurrence frequency	Basic vocabulary roots with interlocking recurrent correspondences
*q’	*k	2x	Seven<br>Louse<br>This/that<br>Earth/land/environment<br>Neck<br>Hand/arm, finger/hand<br>Brush/tree/stick<br>Tongue/cheek<br>Child of woman/son
*q	*k/*g	3x	
*k’	*k	3x	
*k	*k	4x	
*h	*h	2x	
*b’	*ʔ	3x	
*l	*l/r/d	3x	
*ʔ	*ʔ	3x	
*ʔ	*h	2x	

Supplementary Table 8 | Initial comparative dataset of 25 terms analyzed for this study. Whether these similarities are due to cognancy or regularized archaic loans dating to an intense period of interaction prior to the differentiation of the respective protolanguages remains to be determined. PM reconstructions are based on published reconstructions[14]. PC reconstructions are based on multiple studies[9,15–19]

	PM	PC (CU)	PC (H)	PC (P)	Glosses PM/PC
1	*huuq-	*ˈkúh-	—	*kuh	seven/seven
2	*kam	*kap-	*Kap	*kap-	to die/to sleep
3	*ʔab’	—	*hiBA	—	work/work
4	*ʔuk’	*ˈkű	*Ku(N)	*kũʔ	louse/louse
5	*tya	—	—	*^{n}da ~ *ta	generic preposition/locative
6	*kaaʔ	*ˈhákɪ	*haK	*hak ~ *kaʔ ‘stone’	quern/stone (also metate)
7	*haʔ	*hiʔ, *heʔ, *hí/hĩ, *’héʔ ‘that$_{1}$’, *ˈhíʔ	—	*a ‘that’ *hi ~ *iʔ ‘this’	demonstrative base/this one; that one
8	*kab’ ~ *kaab’	*ˈká	*kak	*kaʔ(k) ‘cosmos’	earth, land/place, time, environment, land
9	*qay	*ˈga-	—	*^{ŋ}ga (~ *i’aʔ)	to eat eagerly/to eat, drink
10	*qul ~ *qaal	*ˈgala	—	* ^{ŋ}ga^{n}da	neck/neck
11	*q’ab’	*ˈkU	*k^{w}a	*kuuʔ	hand, arm/finger, digit, hand
12	*k’uul	*ˈkaɾá; *ˈkàr ~ *ka’ri	*kad(a) (~ *kal)	*ka^{n}d- ~ *kat ‘stick, bone, tree’	monte, brush/tree, wood, stick
13	*ŋab’ (PCM)	*ˈdiʔ	*di	*^{n}diʔ	rain/water
14	*paq	*pi	*pi	—	to bend, fold/to fold
15	[*keʔh]	*se/sẽ	*sɨma	*t͜sãĩh	[cold]/(to become) cold
16	*q’iiŋ	*dì, *dui	*dɨw (~ *dɨb), *dɨwi	*^{n}di ‘sun, day’ (cf. *^{n}du- ‘sun, year’)	sun, day/sun, day
17	*taaʔ	*ˈgấ	*ʃa	*^{ŋ}ga	excrement/excrement
18	*t^{y}aq	*diˈsə-	*di-s	*^{n}di^{n}da/*^{n}disa ‘dry$_{1}$’	dry/dry
19	[*ʔaq’]	*aka-	—	—	[to give]/causative
20	*waʔ	*ia (*ya)	—	—	proximal (here, this one)/that one
21	*ʔaaq’ or *ʔaʔq’	*aˈkə	*(h)aka	*haka (~ *akaʔ?)	tongue/sharp, tooth (molar)
22	*ʔar	*ak	—	—	there.is/be (in a place or state)
23	*ʔaal	*aɾa^{2}	—	*La ‘egg, offspring’	child.of.woman/child, offspring
24	*ʔuk’	—	*tuk	*-hu ~ *-uʔ ‘to swallow, drink, eat’	to drink/to drink
25	*kiih	—	—	*ki	Agave/Rope, string

Supplementary Table 9 | Terms for maize in extant and reconstructed Meso- and Central American languages. There is a widely diffused term for ‘maize’[20,21], notated as #ʔayma[22] which we propose can be traced to PC. It is attested in the Lencan family of Honduras and El Salvador, the Misumalpan family of Nicaragua and Honduras, and the Xincan family of Guatemala and formerly Honduras and El Salvador[23]. The term is also present in Mayan. See Supplementary Table 10 for further details.

Language	Term
DIFFUSED ETYMON	#ʔayma[24]
PROTO-MAYAN	*ʔeʔm or Huastec *ʔeem*[25†]
PROTO-CHIBCHAN	*(h)apú or *eb/*ebe or *aiB ~ *aBi, or *aiba[9,18,19†]
PROTO-MISUMALPAN	*ai/*aima[17†]
PROTO-LENCAN	*ayma[26†]
XINCAN	ʔayma[24,27,28]

[†]Proto-language reconstructions

Supplementary Table 10 | Related terms for ‘maize’ (either loans or cognates). Our Chibchan internal differentiation model is based on[9]; for comparison, the II.C. group of Chibchan alone may be as internally diverse as the entire Mayan language family. †ISO-639-3 codes are the most recent standard system for the consistent reference to language names.

**Languages**	**ISO-639-3 Code†**	**‘maize’**
Mayan family		
Huastec[25]	hus	ʔeem
Xincan family[29]		
Xinca[29]	—	ʔayma ‘maize (on cob)’, ʔaʔu ‘maize (degrained)’
Lencan family		
El Salvador	—	ima
Honduras[29]	—	ama
Misumalpan family		
Matagalpa[17,18,30]	mtn	ayma
Cacaopera[17,30]	ccr	ayma
Sumo, Mayangna	yan	ama, am
Ulua, Ulwa[17,30]	ulw	am
Misquito, Mískito, Miskito[17,30]	miq	aya
Chibchan family		
I. Paya, Pech[18]	pay	aʔṹ
II.1.A. Rama[17,19]	rma	ay
II.1.A. Guatuso,Maléku, Maleku[17,19]	gut	ai:ki, a:iŋ
II.B.1.3. Boruca, Borüca, Brunca[17,19]	brn	ep-
II.B.1.1. Bribrig[19]	bzd	i-
II.B.1.1. Cabécar[19]	cjp	i-
II.B.1.2. Naso, Teribe, Térraba, Norteño[17]	tfr	ìb
II.B.2. Dorasque (Chumulu, Gualaca, Changuena)[31]	Qbn, qhi, qqc	hábu, ábu, háu
II.B.3.1. Movere, Guaimí, Ngäbere, Muoi[17,31]	gym	i, heú
II.B.3.1. Bocotá, Bogotá, Buglere[17,32]	sab	eu, íu
II.B.3.2. Cuna, Kuna, Guna, Dulegaya, Cueva[31]	cuk, kvn	om/op(a), <hobba>
II.C.1.1. Muisca, Muysca, Chibcha, Muysccubun[17]	(chb)	aba
II.C.1.2. Tunebo, Uwa[17]	tnd, tbn, tuf, tnb	éba
II.C.1.3. Barí	mot	—
II.C.2.1. Cogui, Kogi[17]	kog	aibi, eibi
II.C.2.1.2.1. Damana, Malayo, Wiwa	mbp	ˈiɨm
II.C.2.1.2.2. Ica, Ika, Bíntucua, Arhuaco[19]	arh	[iʔŋ]
II.C.2.2. Chimila[33]	cbg	á:u ‘maize’

Supplementary Table 11 | AMS radiocarbon dating comparison data for bone collagen and paired enamel samples.

^{14}C ID	Harvard lab ID	Burial ID	Material	Process	^{14}C BP	±	C:N	Cal BP (μ)	Cal BP (2σ lo)	Cal BP (2σ hi)
PSUAMS-1402	I3442	MHCP.14.1.5a	bone, temporal	XAD	4415	25	3.26	4990	5220	4870
PSUAMS-3204	I3442	MHCP.14.1.5a	tooth enamel	enamel carbonate	4250	25	—	4830	4870	4720
PSUAMS-1401	I6235	MHCP.14.1.2a	bone, temporal	XAD	4050	30	3.32	4530	4790	4420
PSUAMS-3202	I6235	MHCP.14.1.2a	tooth enamel	enamel carbonate	3925	25	—	4360	4440	4250
UCIAMS-186360	I5455	MHCP.14.2.4a	tooth, molar	XAD	4005	20	3.29	4480	4530	4420
PSUAMS-3203	I5455	MHCP.14.2.4a	tooth enamel	enamel carbonate	3870	20	—	4310	4420	4230
PSUAMS-1403	I6236	ST.16.1.1	bone, temporal	XAD	4025	25	3.29	4480	4570	4420
PSUAMS-3207	I6236	ST.16.1.1	tooth enamel	enamel carbonate	3740	20	—	4090	4160	3990

Supplementary References

1. Prufer, K. M. & Kennett, D. J. The Holocene Occupations of Southern Belize. in *Approaches to Monumental Landscapes of the Ancient Maya: A Legacy of Human Occupation* (eds. Houk, B., Aroyo, B. & Powis, T.) 16–38 (University of Florida Press, 2020).
2. Asmerom, Y. *et al.* Intertropical convergence zone variability in the Neotropics during the Common Era. *Sci. Adv.* **6**, eaax3644 (2020).
3. Penn, M. G., Sutton, D. A. & Monro, A. Vegetation of the greater Maya mountains, Belize. *Syst. Biodivers.* **2**, 21–44 (2004).
4. Dourson, D. C. *Biodiversity of the Maya Mountains: A Focus on the Bladen Nature Reserve*. (Goatslug Publications, 2012).
5. Piperno, D. R. & Pearsall, D. M. *The origins of agriculture in the lowland neotropics*. (Academic Press, 1998).
6. Kennett, D. J. *et al.* Early Isotopic Evidence for Maize as a Staple Grain in the Americas. *Sci. Adv.* (2020).
7. Prufer, K. M. *et al.* Linking late Paleoindian stone tool technologies and populations in North, Central and South America. *PLOS ONE* **14**, e0219812 (2019).
8. Saul, J. M., Prufer, K. M. & Saul, F. P. Nearer to the gods. Rock shelter burials from the Ek Xux Valley, Belize. in *Stone Houses and Earth Lords: Maya Religion in the Cave Context* 297–323 (University of Colorado Press, 2005).
9. Constenla Umaña, A. Chibchan languages. in *The indigenous languages of South America: A comprehensive guide* 391–439 (De Gruyter Mouton, 2012).
10. Kaufman, T. Aspects of the lexicon of proto-mayan and its earliest descendants. in *The Mayan languages* 62–111 (Routledge Language Family Series, 2017).
11. Monroy Kuhn, J. M., Jakobsson, M. & Günther, T. Estimating genetic kin relationships in prehistoric populations. *PloS One* **13**, e0195491 (2018).
12. Sponheimer, M. & Lee-Thorp, J. A. Alteration of Enamel Carbonate Environments during Fossilization. *J. Archaeol. Sci.* **26**, 143–150 (1999).
13. Posth, C. *et al.* Reconstructing the Deep Population History of Central and South America. *Cell* **175**, 1185-1197 e22 (2018).
14. Kaufman, T. & Justeson, J. Preliminary Mayan Etymological Dictionary. http://www.famsi.org/reports/01051/index.html (2003).
15. Constenla Umaña. Estado actual de la subclasificación de las lenguas chibchenses y de la reconstrucción fonológica y gramatical del protochibchense. *Lingüíst. Chibcha* **XXVII**, 117–135 (2008).
16. Constenla Umaña, A. Comparative Chibchan Phonology. (University of Pennsylvania, 1981).
17. Constenla Umaña, A. ¿Existe relación genealógica entre las lenguas misumalpas y las chibchenses? *Estud. Lingüíst. Chibcha* **XXIV**, 7–85 (2005).
18. Holt, D. G. The development of the Paya sound-system. (University of California at Los Angeles, 1986).
19. Pache, M. Contributions to Chibchan historical linguistics. (Universiteit Leiden, 2018).
20. Campbell, L. Mayan Loan Words in Xinca. *Int. J. Am. Linguist.* **38**, 187–190 (1972).
21. Campbell, L. Cacaopera. *Anthropol. Linguist.* **17**, 146–153 (1975).
22. Kaufman, T. Language History & Language Contact in Pre-Columbian Meso-America. (2020) doi:10.13140/RG.2.2.27129.42081.
23. Campbell, L. *American Indian languages: the historical linguistics of Native America*. (Oxford University Press, 1997).
24. Kaufman, T. MALP 2020. (2020) doi:10.13140/RG.2.2.27129.42081.
25. Kaufman, T. & Justeson, J. Preliminary Mayan Etymological Dictionary. (2003).
26. Cortés, G. R. A. Los fonemas segmentales del protolenca: reconstrucción comparativa. *Rev. Filol. Lingüíst. Univ. Costa Rica* **14**, 89–110 (1988).
27. Campbell, L. Mayan Loan Words in Xinca. *Int. J. Am. Linguist.* **38**, 187–190 (1972).
28. Campbell, L. Distant genetic relationship and the Maya-Chipaya hypothesis. *Anthropol. Linguist.* 113–135 (1973).
29. Sacse, F. *Reconstructive Description of Eighteenth-century Xinka Grammar*. (Utretcht, 2010).
30. Constenla Umaña, A. Acerca de la relación genealógica entre las lenguas lencas y las lenguas misumalpas. *Rev. Filol. Lingüíst.* **XXVIII**, 189–205 (2002).

31. Holt, D. G. The development of the Paya sound-system. (University of California at Los Angeles, 1986).
32. Margery Peña, E. Vocabulario Bocotá. *Estud. Lingüísica Chibcha* **12**, 53–93 (1993).
33. Reichel-Dolmatoff, G. La lengua chimila. *J. Société Américanistes* **36**, 15–50 (1947).
