# Supplementary material for: South-to-north migration preceded the advent of intensive farming in the Maya region
Source: Nat Commun. 2022 Mar 22;13:1530. doi: 10.1038/s41467-022-29158-y (PMC8940966; doi:10.1038/s41467-022-29158-y)
Supplement: Supplementary file 3 — Description of Additional Supplementary Files [file 41467_2022_29158_MOESM3_ESM.pdf]

File Name: Supplementary Data 1
Description: Library information

File Name: Supplementary Data 2
Description: Individual-level contamination estimates

File Name: Supplementary Data 3
Description: Published reference data

File Name: Supplementary Data 4
Description: Outgroup f_3-statistics

File Name: Supplementary Data 5
Description: f_4-statistics comparing 9,600-7,300 BP and 5,600-3,700 BP individuals

File Name: Supplementary Data 6
Description: f_4-statistics testing for allele-sharing between the 9,600-7,300 BP and 5,600-3,700 BP individuals

File Name: Supplementary Data 7
Description: F_4-statistics comparing the 9,600-7,300 BP and 5,600-3,700 BP groups separately to present-day Central and South Americans

File Name: Supplementary Data 8
Description: f_4-statistics comparing 5,600-3,700 BP individuals and present-day Maya

File Name: Supplementary Data 9
Description: newly reported genotype data (zipped file)

Belize.ind	[list of individuals with sex and group ID information]
Belize.snp	[list of SNPs with position information]
Belize.geno	[genotypes]
