# Supplementary material for: South-to-north migration preceded the advent of intensive farming in the Maya region
Source: Nat Commun. 2022 Mar 22;13:1530. doi: 10.1038/s41467-022-29158-y (PMC8940966; doi:10.1038/s41467-022-29158-y)
Supplement: Supplementary file 6 — Reporting Summary [file 41467_2022_29158_MOESM6_ESM.pdf]

nature portfolio

Corresponding author(s): Douglas J. Kennett, Mark Lipson, Keith M. Prufer, David Reich

Last updated by author(s): February 18, 2022

Reporting Summary

Nature Portfolio wishes to improve the reproducibility of the work that we publish. This form provides structure for consistency and transparency in reporting. For further information on Nature Portfolio policies, see our Editorial Policies and the Editorial Policy Checklist.

Statistics

For all statistical analyses, confirm that the following items are present in the figure legend, table legend, main text, or Methods section.

n/a	Confirmed	
☐	☒	The exact sample size (*n*) for each experimental group/condition, given as a discrete number and unit of measurement
☐	☒	A statement on whether measurements were taken from distinct samples or whether the same sample was measured repeatedly
☐	☒	The statistical test(s) used AND whether they are one- or two-sided<br>*Only common tests should be described solely by name; describe more complex techniques in the Methods section.*
☒	☐	A description of all covariates tested
☐	☒	A description of any assumptions or corrections, such as tests of normality and adjustment for multiple comparisons
☐	☒	A full description of the statistical parameters including central tendency (e.g. means) or other basic estimates (e.g. regression coefficient) AND variation (e.g. standard deviation) or associated estimates of uncertainty (e.g. confidence intervals)
☐	☒	For null hypothesis testing, the test statistic (e.g. F, t, r) with confidence intervals, effect sizes, degrees of freedom and P value noted<br>*Give P values as exact values whenever suitable.*
☒	☐	For Bayesian analysis, information on the choice of priors and Markov chain Monte Carlo settings
☒	☐	For hierarchical and complex designs, identification of the appropriate level for tests and full reporting of outcomes
☒	☐	Estimates of effect sizes (e.g. Cohen's d, Pearson's r), indicating how they were calculated

Our web collection on statistics for biologists contains articles on many of the points above.

Software and code

Policy information about availability of computer code

Data collection	BWA v0.6.1, HaploGrep2 v2.1.19, cTools v11, contamMix v1.0-10, ANGSD v0.923, contamLD, other bioinformatics tools and data workflows (https://github.com/DReichLab/ADNA-Tools and https://github.com/DReichLab/adna-workflow), OxCal v4.4
Data analysis	ADMIXTOOLS v7.0.2, EIGENSOFT v7.2.1, hapROH v0.3, QGIS 3.12

For manuscripts utilizing custom algorithms or software that are central to the research but not yet described in published literature, software must be made available to editors and reviewers. We strongly encourage code deposition in a community repository (e.g. GitHub). See the Nature Portfolio guidelines for submitting code & software for further information.

Data

Policy information about availability of data

All manuscripts must include a data availability statement. This statement should provide the following information, where applicable:

- Accession codes, unique identifiers, or web links for publicly available datasets
- A description of any restrictions on data availability
- For clinical datasets or third party data, please ensure that the statement adheres to our policy

The aligned sequences have been deposited in the European Nucleotide Archive database under accession code PRJEB49391 (https://www.ebi.ac.uk/ena/browser/view/PRJEB49391). The processed genotype data used in analysis are available online on the Nature Communications website as a supplementary dataset.

Field-specific reporting

Please select the one below that is the best fit for your research. If you are not sure, read the appropriate sections before making your selection.

☐ Life sciences ☐ Behavioural & social sciences ☒ Ecological, evolutionary & environmental sciences

For a reference copy of the document with all sections, see nature.com/documents/nr-reporting-summary-flat.pdf

Ecological, evolutionary & environmental sciences study design

All studies must disclose on these points even when the disclosure is negative.

Study description	Population genetic analyses were performed on DNA data generated from ancient human skeletons and present-day individuals (previously published). Inferences about historical relationships were made primarily based on allele-sharing patterns across populations, computed using genome-wide SNP genotypes.
Research sample	Twenty one newly reported ancient human individuals buried in present-day Belize, plus 3 previously reported ancient individuals, along with published data from present-day individuals. There are 15 males and 9 females, of which 5 are infants, 3 are old adults, 11 are adults, 3 are sub-adults, and 2 are of indeterminate age. This population represents early Holocene foragers, middle Holocene horticulturalists and late Holocene farmers from the neotropics of Belize.
Sampling strategy	No predetermination was done for the number of individuals. For DNA data generation, we targeted approximately 1.2 million genome-wide SNPs, which effectively cover almost all independent loci (due to linkage disequilibrium) and provide good power in population history analyses.
Data collection	DNA from the ancient skeletons was extracted, sequenced, and processed into SNP genotypes (final data overseen by D.R.).
Timing and spatial scale	Ancient individuals with burial dates of ~9,600-3,700 years ago were sampled during five field seasons of excavations between 2014 and 2019 at two rock-shelter sites in Belize. The rock-shelters are located ~1.8km apart in the remote Bladen Nature Reserve in the Maya Mountains of Belize. Fieldwork in remote Bladen Nature Reserve is only possible during the dry season (March-May) due to extreme weather hazards during the rainy season.
Data exclusions	We assessed data quality via standard ancient DNA criteria and excluded individuals with either insufficient sequencing coverage or evidence of contamination.
Reproducibility	All attempts to reproduce were successful.
Randomization	Analyses were performed either on an individual basis or in two chronological subgroups (based on the observed similarity of individuals within each).
Blinding	Analyses were performed similarly for all individuals.

Did the study involve field work? ☒ Yes ☐ No

Field work, collection and transport

Field conditions	Both caves (rockshelters) are located on karst limestone in the Maya Mountains of Belize. Mean annual temperature is 26 degrees C and mean annual rainfall exceeds 4000mm and is seasonally distributed. Vegetation in the study area is composed of broad leaf tropical forest.
Location	LAT16.5°/LON-88.9°, 480 MASL
Access & import/export	Field research conducted under permits issued to KM Prufer by the Belize Inst. of Archaeology and Belize Forest Dept and overseen by Ya'axche Conservation trust. All materials exported by bonded carrier to the University of New Mexico under permits issued to KM Prufer by the Belize Inst. of Archaeology.
Disturbance	Excavations were backfilled upon completion. Camping followed "leave no trace" protocols to minimize impacts.

Reporting for specific materials, systems and methods

We require information from authors about some types of materials, experimental systems and methods used in many studies. Here, indicate whether each material, system or method listed is relevant to your study. If you are not sure if a list item applies to your research, read the appropriate section before selecting a response.

Materials & experimental systems

n/a	Involved in the study
☒	☐ Antibodies
☒	☐ Eukaryotic cell lines
☐	☒ Palaeontology and archaeology
☒	☐ Animals and other organisms
☒	☐ Human research participants
☒	☐ Clinical data
☒	☐ Dual use research of concern

Methods

n/a	Involved in the study
☒	☐ ChIP-seq
☒	☐ Flow cytometry
☒	☐ MRI-based neuroimaging

Palaeontology and Archaeology

Specimen provenance

All samples were mapped, plotted, and removed during stratigraphic excavations at depths between 20cm and 320cm below excavation datums. Charcoal samples used to date three individuals were also plotted during excavations. All ancient skeletons from the MHCP and ST rock-shelters were excavated by the Bladen Paleoindian and Archaic Archaeological Project (BPAAP), directed by KM Prufer and DJ Kennett.

Specimen deposition

All samples are curated in the Maxwell Museum and Department of Anthropology at the University of New Mexico.

Dating methods

We directly radiocarbon (14C) dated all newly reported individuals via accelerator mass spectrometry (AMS) (Supplementary Table 11). Bone collagen yields were generally low from these depositional contexts, and multiple extractions were required for each sample to obtain datable material. In most instances, extracted collagen was hydrolyzed and amino acids were purified using solid phase extraction columns (XAD amino acids). Crude gelatin yields were high enough for one sample (MHCP.17.1.7) to use a modified Longin method with ultrafiltration. The preservation of extracted and purified collagen or amino acid samples was evaluated using crude gelatin yields (% wt) and stable carbon and nitrogen isotope mass spectrometry (%C, %N and C/N ratios; Thermo DeltaPlus with a Costech elemental analyzer at Yale University). C/N ratios between 3.22 and 3.40 indicate that all radiocarbon dated collagen and amino acid samples were well preserved. We directly dated enamel carbonate in four samples after multiple failed attempts at extracting collagen or amino acids. Carbonate samples were chemically cleaned using published procedures, and sample integrity was evaluated using Fourier-transform infrared spectroscopy (FTIR, Supplementary Table 11, Supplementary Fig. 24) and stable isotope mass spectrometry. To assess AMS 14C dated enamel we processed paired collagen samples from the same individuals and determined that the enamel dates were ~125-285 years younger than the paired collagen date (Supplementary Fig. 25), but we have not corrected the original dates in this analysis. After quality assurance all samples were combusted (collagen and amino acids) or hydrolyzed (carbonate) and graphitized at Penn State University (PSU). 14C measurements were made on a modified National Electronics Corporation compact spectrometer at either PSUAMS or UCIAMS radiocarbon facilities. All dates were calibrated in OxCal version 4.481 using the IntCal20 curve82 and are presented in calendar years before present (cal. BP). Multiple radiocarbon dates on the same individual were combined using the R_Combine command in OxCal.

☒ Tick this box to confirm that the raw and calibrated dates are available in the paper or in Supplementary Information.

Ethics oversight

All ancient skeletons from the MHCP and ST rock-shelters were excavated by the Bladen Paleoindian and Archaic Archaeological Project (BPAAP) under permits issued by the Belize Institute of Archaeology (IA) and the Belize Forest Department. Skeletons of ancient individuals were exported under permits issued by the IA in accordance with the laws of Belize and permission granted to conduct molecular analyses on bulk tissues extracted from skeletons of ancient individuals. Research was conducted in close collaboration with the Ya'axché Conservation Trust, an internationally recognized Belizean NGO that is the co-manager of the Bladen Nature Reserve (BNR) with the Government of Belize. Ya'axché is locally managed and largely staffed by members of descendant Maya communities. We engaged in formal consultations with descendant communities as described in the article.

Note that full information on the approval of the study protocol must also be provided in the manuscript.
